# Prenatal exposure to benzo[a]pyrene depletes ovarian reserve and masculinizes embryonic ovarian germ cell transcriptome transgenerationally

**DOI:** 10.1038/s41598-023-35494-w

**Published:** 2023-05-29

**Authors:** Jinhwan Lim, Toshihiro Shioda, Kelli F. Malott, Keiko Shioda, Junko Odajima, Kathleen N. Leon Parada, Julie Nguyen, Samantha Getze, Melody Lee, Jonathon Nguyen, Samantha Reshel Blakeley, Vienna Trinh, Hong-An Truong, Ulrike Luderer

**Affiliations:** 1 Department of Environmental and Occupational Health, University of California, Irvine (UCI), Irvine, CA 92617 USA; 2 Massachusetts General Center for Cancer Research and Harvard Medical School, Charlestown, MA 02129 USA; 3 Environmental Health Sciences Graduate Program, UCI, Irvine, CA 92617 USA; 4 Department of Developmental and Cell Biology, UCI, Irvine, CA 92617 USA; 5 Department of Medicine, UCI, Irvine, CA 92617 USA; 6 Center for Occupational and Environmental Health, 856 Health Sciences Rd, Suite 3200, Zot 1830, Irvine, CA 92697 USA

**Keywords:** Developmental biology, Endocrinology

## Abstract

People are widely exposed to polycyclic aromatic hydrocarbons, like benzo[a]pyrene (BaP). Prior studies showed that prenatal exposure to BaP depletes germ cells in ovaries, causing earlier onset of ovarian senescence post-natally; developing testes were affected at higher doses than ovaries. Our primary objective was to determine if prenatal BaP exposure results in transgenerational effects on ovaries and testes. We orally dosed pregnant germ cell-specific EGFP-expressing mice (F0) with 0.033, 0.2, or 2 mg/kg-day BaP or vehicle from embryonic day (E) 6.5–11.5 (F1 offspring) or E6.5–15.5 (F2 and F3). Ovarian germ cells at E13.5 and follicle numbers at postnatal day 21 were significantly decreased in F3 females at all doses of BaP; testicular germ cell numbers were not affected. E13.5 germ cell RNA-sequencing revealed significantly increased expression of male-specific genes in female germ cells across generations and BaP doses. Next, we compared the ovarian effects of 2 mg/kg-day BaP dosing to wild type C57BL/6J F0 dams from E6.5–11.5 or E12.5–17.5. We observed no effects on F3 ovarian follicle numbers with either of the shorter dosing windows. Our results demonstrate that F0 BaP exposure from E6.5–15.5 decreased the number of and partially disrupted transcriptomic sexual identity of female germ cells transgenerationally.

## Introduction

Polycyclic aromatic hydrocarbons (PAHs) are products of incomplete combustion of fossil fuels, wood, tobacco, food and other organic materials^1^. People are exposed to PAHs largely via inhalation of particulate matter air pollution and tobacco smoke and via consumption of grilled or smoked foods^1–3^. Human biomonitoring data demonstrate that essentially all people are exposed to PAHs^4^. The PAH benzo[a]pyrene (BaP) is composed of five fused aromatic rings^1^. BaP, like other PAHs, undergoes metabolism to reactive metabolites, which are largely responsible for its toxicity^5^. Metabolic pathways include oxidation by cytochrome P450s, yielding epoxides, followed by epoxide hydrolase-mediated conversion to dihydrodiols. The latter can undergo further metabolism to highly reactive diol epoxides and *o*-quinones, which react with DNA forming bulky adducts^6–8^. The *o-*quinones can undergo redox cycling, resulting in formation of reactive oxygen species^7,8^. Another pathway, in which BaP acts as a reducing substrate for peroxidases, yields BaP radical cations, which are also DNA reactive^9^. Several BaP metabolites are aryl hydrocarbon receptor agonists and others are estrogen receptor agonists^10,11^. Thus exposure to BaP may result in toxic effects by inducing DNA damage or by activating arylhydrocarbon or estrogen receptors to initiate aberrant signaling.

In utero exposure to BaP dose-dependently decreased fertility of male and female offspring, with complete sterility observed in both sexes with doses of 40 mg/kg-day and higher on embryonic days (E) 7–16^12^. Females are born with a finite number of oocytes, which become surrounded by specialized somatic cells called granulosa cells, forming primordial follicles during the first postnatal week in mice and during the second trimester in humans^13^. Any exposure that decreases the size of the primordial follicle pool, often referred to as the ovarian reserve, will result in early onset of reproductive senescence in females. In contrast, males possess germline stem cells throughout life. Ovarian follicle numbers and testicular spermatid numbers were dose-dependently decreased following in utero exposure to 2 and 10 mg/kg-day BaP from E6.5–15.5, with significant effects observed at the lower dose in the F1 female offspring, but only at the higher dose in the F1 males^14,15^. Embryos deficient in the antioxidant and Phase 2 biotransformation cofactor glutathione were more sensitive to the in utero gonadal toxicity of BaP than wild type littermates^14,15^. BaP^16,17^ and another PAH, 9,10-dimethylbenz[a]anthracene^18,19^, induced apoptosis in germ cells of cultured E13.5 ovaries, but not E13.5 testes^16^, via BAX mediated caspase activation. Consistent with greater sensitivity of the developing ovary than the developing testis to BaP, the E13.5 ovarian transcriptome was significantly affected by BaP exposure, while the testicular E13.5 transcriptome was not significantly affected^20^.

In recent years there has been growing concern that in utero exposure to chemical toxicants can cause adverse health outcomes not only in the developing embryo of an exposed F0 pregnant female (F1 offspring), but also in their F2 daughters exposed as germ cells in the developing F1 female and their unexposed F3 grand-daughters^21–24^. Similarly, exposures of males to chemical toxicants have been shown to cause heritable effects in their offspring^21–24^. Effects are called multigenerational if they occur in the exposed F1 and F2 offspring of exposed F0 pregnant females or in the F1 offspring of exposed F0 males. They are considered transgenerational if they occur in the F3 or later generations descended from exposed F0 females or in the F2 and later generations descended from exposed F0 males. Several epigenetic mechanisms have been proposed for transgenerational inheritance including alterations in DNA methylation, histone modifications, and noncoding RNAs^21–25^. Mohamed et al.^26^ demonstrated F2 effects of F0 male dosing with 0, 1, or 10 mg/kg-day BaP orally for 6 wk prior to mating to an untreated female, followed by mating of F1 and F2 males with unexposed females to generate F2 and F3 males. They reported significantly decreased numbers of seminiferous tubules having elongated spermatids, as well as decreased epididymal sperm counts in F0, F1, and F2 males, and no effects in F3 males^26^. To our knowledge this is the only published study that investigated transgenerational effects of any PAH.

In the mouse, the primordial germ cell (PGC) precursors form in the proximal epiblast around E6.0–6.5, become specified to PGCs around E7.25^27^, proliferate and begin migrating along the hindgut mesentery to the gonadal ridges, where they continue to proliferate until beginning to enter meiosis in the female at E13.5 (now called oocytes) and undergoing mitotic arrest in the male at E13.5^28^. E13.5 is also the time point during development when the germ cell DNA is maximally demethylated in both sexes^29–31^. The male E13 and E16 primordial germ cell transcriptomes were reportedly altered in a transgenerational manner after in utero exposure to the fungicide vinclozolin^32,33^. No prior studies of which we are aware have reported on the effects of in utero exposure to any environmental toxicant on the ovarian PGC transcriptome.

We hypothesized that in utero exposure to BaP results in diminished ovarian reserve not only in the F1 female offspring, but also in the F2 and F3 females and that this is associated with transgenerational alterations in the E13.5 ovarian PGC transcriptomes. Further, we hypothesized that males are less sensitive to the transgenerational gonadal effects of in utero exposure to BaP than females*.* Finally, we hypothesized that the mitotic window of ovarian development is more sensitive than the predominantly meiotic window of ovarian development to direct and transgenerational effects of in utero exposure to BaP.

## Results

### F0 exposure to BaP transgenerationally decreases the ovarian reserve

**Figure 1:**
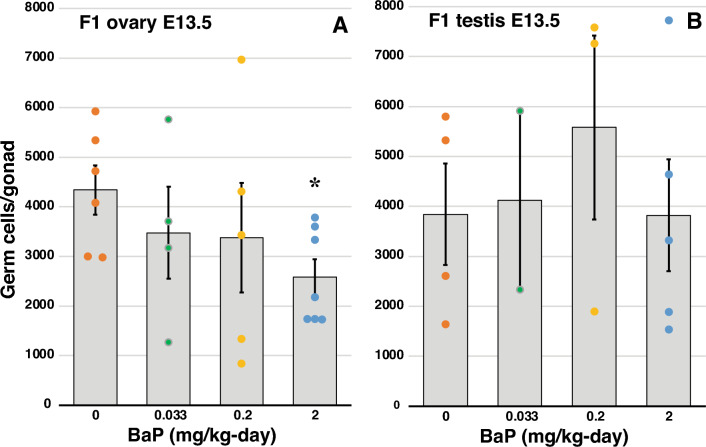
Effects of F0 dam exposure to BaP on germ cell numbers in gonads of F1 embryos (Experiment 1). Pregnant F0 OG2 females were exposed to 0.033, 0.2, or 2 mg/kg-day BaP or vehicle from E6.5–11.5, and F1 embryo gonads were collected at E13.5 for germ cell counts. Graphs show means ± SEM of total number of germ cells per gonad. Dots represent values for individual embryos. (**A**) The number of ovarian germ cells was significantly decreased in BaP-exposed compared to oil controls (*P* = 0.100, effect of BaP dose, linear regression; *P = 0.014, 2 versus 0 mg/kg-day BaP, t-test). (**B**) The number of F1 testicular germ cells was not affected by F0 BaP exposure (*P* = 0.687, effect of BaP dose, linear regression). N = 2–7 F1 embryos/group, each from independent F0 dam.

**Figure 2:**
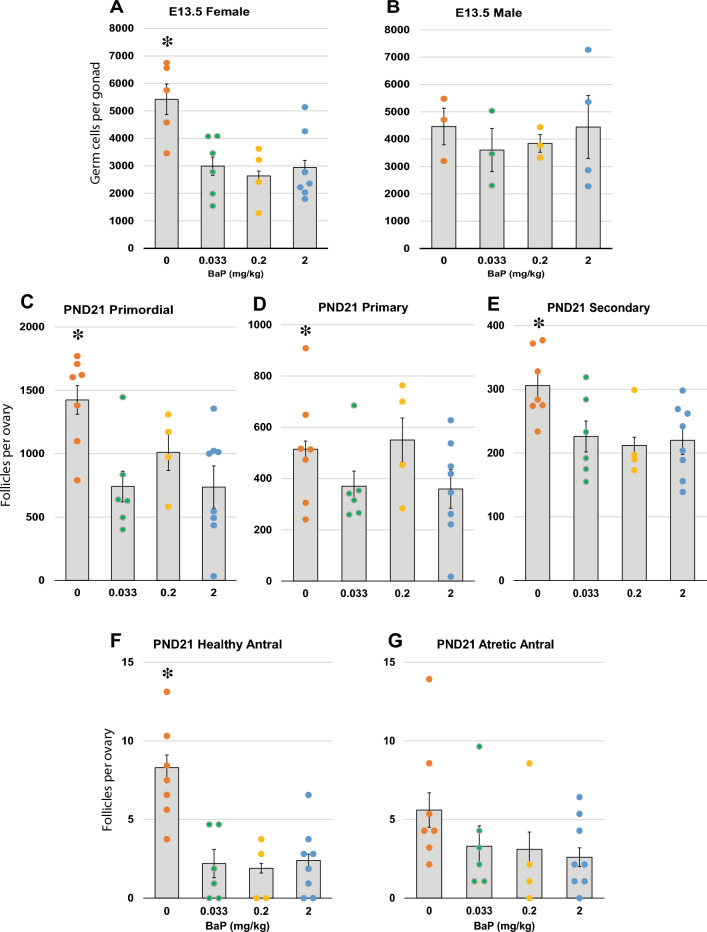
Effects of F0 dam exposure to BaP on germ cell and follicle numbers in gonads of F3 offspring (Experiment 1). Pregnant F0 OG2 females were exposed to 0, 0.033, 0.2, or 2 mg/kg-day BaP from E6.5–15.5. F3 embryonic gonads were collected at E13.5 and F3 ovaries were collected at PND21 for germ cell and follicle counts, respectively. Graphs show estimated marginal means ± SEM of total number of germ cells or follicles per gonad from GEE models. Dots represent values for individual embryos. (**A**) The number of germ cells was significantly decreased in the F3 E13.5 ovaries (A; *P < 0.001, beta for BaP dose = 0 mg/kg compared to other doses, GEE), but not in the F3 E13.5 testes (**B**) of mice descended from BaP-exposed compared to control F0 dams. The numbers of primordial (**C**; *P < 0.001, beta for BaP dose = 0 mg/kg compared to other doses, GEE), primary (**D**; *P = 0.021, beta for BaP dose = 0 mg/kg compared to other doses, GEE), secondary (**E**; *P = 0.004, beta for BaP dose = 0 mg/kg compared to other doses, GEE), and healthy antral (**F**; *P < 0.001, beta for BaP dose = 0 mg/kg compared to other doses, GEE) follicles were significantly decreased in F3 PND21 ovaries of mice descended from BaP-exposed compared to control F0 dams (*P* < 0.03, effect of BaP dose, GEE), while atretic antral follicle numbers were not significantly decreased (**G**). N = 4–7 F3 PND21 females derived from 3 to 5 F0 dams per group.

We previously reported that oral dosing of pregnant F0 dams with 2 or 10 mg/kg-day BaP from E6.5–15.5 significantly and dose-dependently decreased ovarian reserve in F1 offspring^14^. The POU transcription factor OCT4, also known as POU5F1, is expressed in proliferating PGCs, is downregulated when oocytes initiate meiosis, and is expressed again in oocytes from the primordial follicle stage onward^34^. We utilized mice expressing Enhanced Green Fluorescent Protein under control of *Oct4* promoter and enhancers (OG2 mice)^35^ as a model system to investigate the effects of in utero exposure to BaP during critical windows of gonadal development, from E6.5 to E17.5, on germ cell numbers and on the E13.5 PGC transcriptomes in F1, F2, and F3 generations. We orally dosed pregnant F0 OG2 dams with 0.033, 0.2, or 2 mg/kg-day from E6.5–11.5 for collection of F1 ovaries and testes at E13.5 and from E6.5–15.5 for collection of F3 gonads at E13.5 and PND21. F0 exposure to 2 mg/kg-day BaP from E6.5–11.5 resulted in a statistically significant decrease in ovarian germ cell numbers in F1 females exposed prenatally to 2 mg/kg-day BaP at E13.5 (*P* = 0.10, effect of BaP by linear regression; *P* = 0.014, t-test, 2 mg/kg-day versus 0 mg/kg-day; Fig. 1A), while having no effect on testicular germ cell numbers in F1 males at E13.5 (*P* = 0.687, effect of BaP by linear regression; Fig. 1B). F0 BaP exposure from E6.5–15.5 resulted in statistically significantly decreased germ cell numbers in F3 female embryos at E13.5 (Fig. 2A; *P* < 0.001, beta for BaP dose = 0 mg/kg compared to other doses, Generalized Estimating Equation, GEE), but not in F3 E13.5 males (Fig. 2B). We confirmed this transgenerational effect on the ovarian reserve at PND 21, when F3 primordial, primary, secondary, and antral follicles were significantly decreased by ancestral BaP exposure compared to ancestrally vehicle exposed control F3 mice (Fig. 2B–F; *P* < 0.03, effect of BaP dose, GEE).

**Figure 3:**
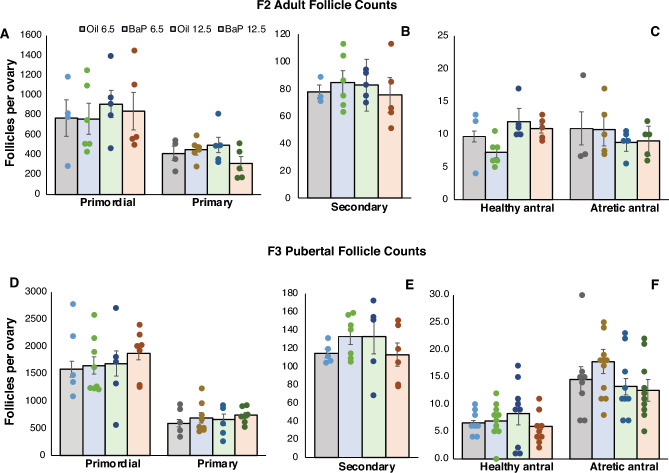
Effects of F0 dam exposure to BaP during the mitotic or meiotic windows of ovarian development on ovarian follicle counts in their F2 and F3 offspring (Experiment 2). Pregnant C57BL/6J females were exposed to BaP or oil vehicle from E6.5–11.5 (mitotic developmental window) or E12.5–17.5 (predominantly meiotic developmental window). Adult, 4 to 5 month old F2 female offspring were euthanized on the day of estrus of the estrous cycle based on vaginal cytology (N = 4–6 F2 females derived from 4–5 F0 females/group). F3 female offspring were euthanized on the day of first vaginal estrus (puberty) for ovarian follicle counts (N = 9–11 F3 females derived from 5 to 7 F0 dams/group). Graphs show estimated marginal means ± SEM of total number of follicles of different developmental stages per gonad from GEE models. Dots represent values for individual females. There were no statistically significant effects of F0 BaP exposure, developmental window, or interaction between BaP and developmental window on primordial (**A**,**D**), primary (**A**,**D**), secondary (**B**,**E**), healthy antral (**C**,**F**), or atretic antral (**C**,**F**) follicle numbers in F2 or F3 ovaries.

To test the hypothesis that developing ovaries are more sensitive to the transgenerational effects of BaP on the ovarian reserve during germ cell specification and proliferation than during meiosis initiation and arrest, we compared the effects of oral BaP dosing of pregnant C57BL/6J dams with 0 or 2 mg/kg-day from E6.5–11.5 (PGC mitotic window) with dosing from E12.5–17.5 (meiotic window) on ovarian follicle numbers in F2 and F3 females. We previously reported statistically significantly decreased primordial, primary, and secondary follicle numbers in BaP-exposed F1 females from this experiment at puberty, defined as first vaginal estrus, with no effect of exposure window^36^. However, in contrast to the results with F0 BaP dosing from E6.5–15.5, we observed no statistically significant effects of F0 BaP treatment on ovarian follicle numbers in the adult F2 or pubertal F3 females descended from F0 mice dosed during either of the shorter mitotic or meiotic developmental windows (Fig. 3).

In a third experiment, using mice heterozygous for Glutamate Cysteine Ligase Modifier subunit (*Gclm*, the modifier subunit of the rate-limiting enzyme in glutathione synthesis), we found that primordial, primary, and secondary follicles per ovary were significantly decreased in 4–5 month old F2 *Gclm*+*/−* mice descended from *Gclm*+*/−* F0 dams dosed from E6.5–15.5 with 2 mg/kg-day BaP compared to F2 mice descended from control F0 dams (Fig. S1).

### Transgenerational impact of gestational F0 exposure to BaP involved disruption of transcriptomic sex identities of E13.5 primordial germ cells

**Figure 4:**
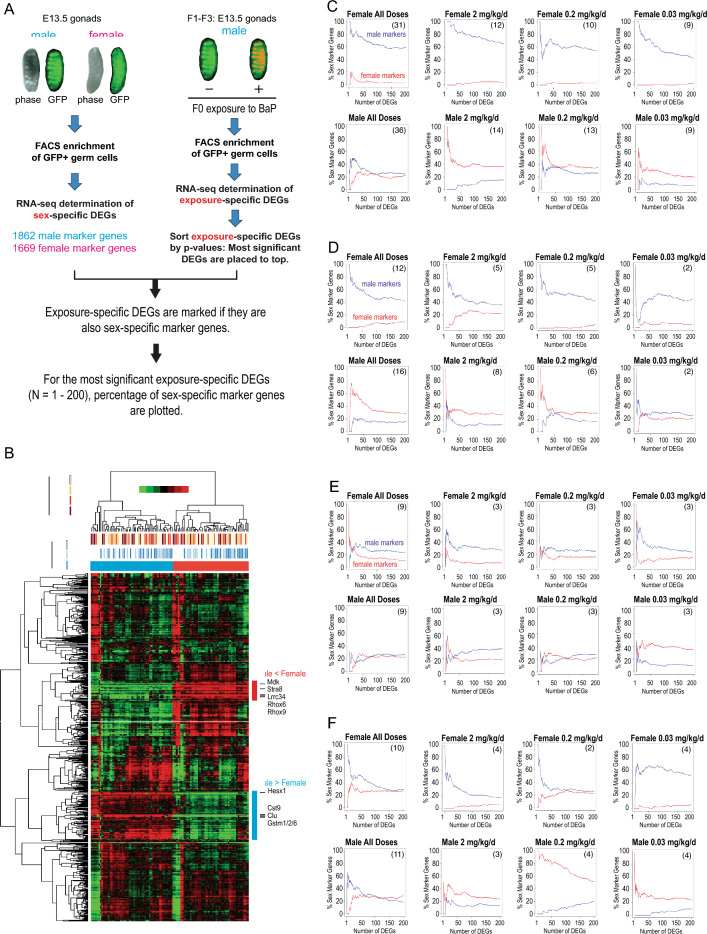
Enrichment of gonadal sex-specific differentially expressed genes (DEGs) in BaP exposure-specific DEGs. (**A**) Analysis flow chart. Primordial germ cells (PGCs) isolated from E13.5 embryonic gonads of OG2 descended from pregnant, vehicle-exposed F0 mice were subjected to RNA-seq to determine sex-specific DEGs (left column). RNA-seq data of PGCs isolated from F1–F3 E13.5 male gonads with F0 exposure to BaP or F0 exposure to vehicle from E6.5–11.5 (F1) or E6.5–15.5 (F2, F3) were examined to obtain *p*-value ranked lists of exposure-specific DEGs (right column). The same procedures were performed for the RNA-seq data of PGCs isolated from F1-F3 female gonads (not shown). Numbers of sex-specific DEGs within the exposure-specific DEGs were calculated for up to 200 exposure-specific DEGs with the smallest *p*-values. (**B**) Heatmap of unsupervised hierarchical clustering of PGC transcriptomes. Locations of sex-specific DEGs are shown by colored bars on the right with representative DEGs. (**C**, **D**, **E**) Enrichment of sex-specific DEGs in the exposure-specific DEGs calculated for PGCs in (**C**) all generations of embryos, (**D**) PGCs in the F1 embryos, (**E**) PGCs in the F2 embryos, or (**F**) PGCs in the F3 embryos. Y-axis shows relative frequencies of sex-specific genes (blue and red lines show male- and female-specific genes, respectively). X-axis shows numbers of exposure-specific genes with smallest *p*-values. Numbers of exposed embryos examined for each panel are shown in parentheses in upper left. Numbers of embryos isolated from vehicle exposed control animals were as follows: 17 males and 17 females for panel **C**, 5 males and 5 females for panel **D**, 6 males and 6 females for panel **E**, and 6 males and 6 females for panel **F**.

Our recently published study showed that gestational exposures of pregnant C57BL/6J female mice to BaP sex-dependently impacted the E13.5 gonadal transcriptome, with 490 significantly differentially expressed genes (False Discovery Rate, FDR, *p*-value < 0.05) in the E13.5 ovaries and none in the E13.5 testes^20^. In the present study we attempted to examine whether in utero exposure of mouse embryos to BaP affects sex differentiation of primordial germ cells (PGCs). In mice, PGCs arrive at the gonadal ridge E10.5^37^, where they proliferate until entering meiosis in females starting at E13.5 (now called oocytes), while in males they proliferate, migrate to the forming seminiferous cords (now called gonocytes)^38^, and undergo mitotic arrest shortly before birth^38^. Herein we refer to E13.5 germ cells as PGCs in both sexes as a shorthand. To collect E13.5 mouse PGCs, we took advantage of the OG2 transgenic mice^35^, which express EGFP from the germline-specific *Pou5f1* promoter, as we previously described^39^. OG2 homozygous females were timed-mated with OG2 homozygous males. At E13.5, when PGCs are settled in the embryonic gonads and have initiated sexual differentiation as dictated by the surrounding gonadal somatic cells^40^, EGFP-expressing PGCs (Fig. 4A, top images) were isolated by FACS and subjected to RNA-seq. Representative FACS data demonstrate purity of OCT4-EGFP positive cells (Fig. S2). Normalized counts of mRNA expression showed no sign of batch effects by generation, sex, or exposure (Fig. S3).

BigWig tracks of RNA-seq data pooled for BaP dosage, gonadal sex, and generations (Fig. S4) showed clear differential expression of representative germline sex marker genes—namely, the male marker gene *Lhx1* was far more strongly expressed in male PGCs than female PGCs whereas the female germline marker *Stra8* was expressed exclusively in female PGCs. The absolute lack of mRNA expression from the constitutively active, Y-chromosomal gene *Eif2s3y* in female PGCs confirmed the absence of any contaminating male cells in female PGC specimens. The PGC marker gene *Nanos3* and markers of later-stage germline cells *Ddx4* and *Dazl* were expressed at comparable levels between male and female PGCs in all generations, confirming the germline nature of the FACS-isolated PGC specimens.

We initiated our transcriptomic analysis of PGCs with unsupervised hierarchical clustering of RNA-seq data. A heatmap representation of the clustering analysis showed clear separation of male and female PGCs (Fig. 4B), indicating a significant degree of sexual differentiation of the germline cells in the E13.5 embryonic gonadal anlage, and several known germline sex marker genes are recognized as differentially expressed genes (DEGs; colored bars on the right of the heatmap). However, exposure doses (0, 0.03, 0.2, or 2 mg/kg/d) or generations of the embryos (F1, F2, or F3) did not form significant clusters, indicating that gestational F0 exposure did not cause readily detectable strong transcriptomic impact on E13.5 PGCs of the subsequent generations.

Next, we attempted to examine whether exposure-specific DEGs provide us with clues to disturbed sexual differentiation of E13.5 PGCs by gestational F0 exposure to BaP. Firstly, we determined marker genes of sexual differentiation of E13.5 PGCs by RNA-seq and identified 1862 and 1669 DEGs specifically expressed in male and female PGCs from vehicle control lineages, respectively (> twofold increase in one sex compared to the other, FDR < 0.05; N = 17 for each sex; Fig. 4A, left column, and Table S1). The numbers of our sex-specific DEGs were 3–fourfold greater than those previously reported by Sakashita et al*.*^41^ who determined sex-specific DEGs in E13.5 mouse PGCs (C57BL/6 background) taking a similar approach and the same set of DEG criteria as ours but with limited numbers of biological replicates (N = 2 for each sex). Secondly, we identified 3100 genes whose expression counts exceeded 100 in any E13.5 PGCs in any generations of embryos (F1–F3) with or without F0 exposure to BaP (Table S2). Thirdly, we determined exposure-specific DEGs in E13.5 PGCs isolated from F1–F3 embryos and sorted DEGs by statistical significance so that DEGs with smallest *P*-value are ranked to the top (Fig. 4A, right column, and Tables S3–S6).

After the list of sex-specific DEGs, exposure-specific DEGs, and significantly expressed genes had been generated, we attempted to integrate the sex-specific DEGs and the exposure-specific DEGs. To prepare for this analysis, we performed permutation tests to establish the probabilities of accidentally observing sex-specific DEGs in a randomly sampled list of significantly expressed genes (Fig. S5). For example, when 50 genes (Sampling Size = 50) were randomly selected, some of them—typically 4–5% of the 100 genes—were in the list of sex-specific DEGs. By repeating this sampling 5000 times, we generated histograms representing the distribution of probabilities of observing sex-specific DEGs in the list of randomly chosen genes. Gamma-distribution fitting demonstrates that the distribution was strongly biased towards smaller numbers when the sample size was 50 and that the distribution became more symmetrical centering at 5% as the Sample Size increased to 100 and 200. The results of these permutation tests demonstrate that the probability that sex-specific DEGs occupy more than 10% of the randomly chosen expressed genes was statistically significant (*P* < 0.05) regardless of the sample size.

We then determined numbers of the sex-specific DEGs in varying numbers of the statistically most significant exposure-specific DEGs and calculated the percentages of sex-specific DEGs per 1–200 exposure-specific DEGs (Fig. 4A,C–F). When data of all generations and all doses were combined for the above analysis (Fig. 4C, “Female All Doses”), the exposure-specific DEGs in female PGCs showed strong enrichment of the male sex-specific DEGs (blue line) for all numbers of the exposure-specific DEGs tested whereas the female-specific DEGs (red line) were not enriched. Strong enrichment of the male sex-specific DEGs was also evident for all doses of F0 BaP exposure (Fig. 4C, “Female 2, 0.2, or 0.033 mg/kg/d”). In contrast, both male and female exposure-specific DEGs in male PGCs showed an only modest degree of enrichment (Fig. 4C, “Male All Doses”), although the largest F0 BaP exposure caused enrichment of the female exposure-specific DEGs (red line in “Male 2 mg/kg/d”). Taking the same approach, we examined transcriptomes of PGCs isolated from the F1, F2, and F3 embryos. Female PGCs in the F1 embryos (Fig. 4D) again showed enrichment of the male sex-specific DEGs after the 2 and 0.2 mg/kg/d F0 exposure. The female sex-specific DEGs were not enriched except for modest enrichment with the high dose exposure (2 mg/kg/d). Male F1 PGCs showed modest degrees of enrichment of both male and female sex-specific DEGs although female-specific DEGs tended to be more strongly enriched. Female F2 PGCs showed enrichment, albeit less pronounced than in F1, of male-specific genes (Fig. 4E). Female F3 PGCs in the F3 embryos showed strong enrichment of the male sex-specific DEGs after the 2 and 0.033 mg/kg/d F0 exposure whereas female sex-specific DEGs were not enriched (Fig. 4F). When all doses were combined, female F3 PGCs show modest enrichment of male- and female-specific DEGs. Male F3 PGCs showed modest enrichment of both male and female sex-specific DEGs at all doses except that the low dose (0.033 mg/kg/d) F0 exposure did not cause enrichment of male-specific DEGs. The high and low F0 exposure doses tended to enrich female-specific DEGs in male F3 PGCs more strongly than male-specific DEGs. These observations collectively support the notion that gestational exposure of F0 mothers to BaP caused transgenerationally transmitted disturbance in transcriptomic sexual differentiation of E13.5 PGCs.

### Gene ontology (GO) analyses of DEGs in PGCs of F1, F2, and F3 offspring directly or ancestrally exposed to BaP

As noted above, using the stringent criterion of FDR p-value, we observed few statistically significant DEGs when comparing BaP-exposed to vehicle-exposed PGCs in any generation. Specifically, by this criterion, we observed 0, 1, and 7 DEGs, respectively, in the F1 0.033. 0.2, and 2.0 mg/kg-day BaP-exposed compared to oil exposed females; none in the F2 BaP-exposed compared to oil-exposed females, and 25, 6, and 3 DEGs, respectively, in the F3 0.033. 0.2, and 2.0 mg/kg-day ancestrally BaP-exposed compared to oil exposed females (Tables S4, S5, and S6). In the F1 males we observed no DEGs with any BaP dose; we observed 0, 78, and 3 DEGs, respectively, in the F2 0.033. 0.2, and 2.0 mg/kg-day BaP-exposed compared to oil-exposed males, and 0, 5, and 0 DEGs, respectively, in the F3 0.033. 0.2, and 2.0 mg/kg-day ancestrally BaP-exposed compared to oil exposed males (Tables S4, S5, and S6).

To better understand which biological processes may be driving the transgenerational sex disturbances, we used less stringent criteria for inclusion of DEGs in Gene Ontology (GO) analyses using GOrilla^42^. Specifically, we used lists of DEGs, which were 1.5-fold or more up or down-regulated and had unadjusted *p*-values < 0.05, as the target genes against the background list of all 15,423 expressed genes. We then used Revigo to remove redundant GO terms^43^.

No GO terms that were statistically significant by FDR p-value were identified by GOrilla in females from the F1 2 mg/kg-day or 0.033 mg/kg-day, any F2 dose groups, or F3 2 mg/kg-day and 0.2 mg/kg-day groups compared to oil controls.

For F1 0.2 mg/kg-day exposed females compared to oil controls, GOrilla identified 21 GO terms, of which 18 were non-redundant and two had statistically significant FDR p-values (inflammatory response and defense response). For the F3 0.033 mg/kg-day exposed females compared to control females, GOrilla identified 157 GO terms, of which 108 were non-redundant and 88 had statistically significant FDR p-values and of which many related to immune, inflammatory, and defense responses (Table S7).

No GO terms that were statistically significant by FDR p-value were identified by GOrilla in males of the F1 2 mg/kg-day, F1 0.2 mg/kg-day, F2 0.033 mg/kg-day, or F3 0.033 mg/kg-day groups compared to oil controls.

For F1 0.033 mg/kg-day exposed males compared to oil controls, GOrilla identified 45 GO terms, of which 40 were non-redundant and of which 14 had statistically significant FDR p-values (Table S8). Significant GO terms included myeloid leukocyte chemotaxis and defense response.

For F2 2 mg/kg-day exposed males compared to oil controls, GOrilla identified 82 GO terms, of which 61 were non-redundant and of which 37 had statistically significant FDR p-values (Table S9). For F2 0.2 mg/kg-day exposed males compared to oil controls, GOrilla identified 88 GO terms, of which 57 were non-redundant and of which 14 had statistically significant FDR p-values (Table S10). Significant GO terms included leukocyte chemotaxis, immune response, humoral immune response and defense response.

For F3 0.2 mg/kg-day exposed males compared to oil controls, GOrilla identified 156 GO terms, of which 118 were non-redundant and of which 102 had statistically significant FDR *p*-values (Table S11). For F3 2 mg/kg-day exposed males compared to oil controls, GOrilla identified 15 GO terms, of which 13 were non-redundant and of which 2 had statistically significant FDR p-values (gas transport; oxygen transport).

### Gestational F0 exposure to BaP increased mRNA and protein expression of the male PGC genes *Tdgf1* and *Lefty2* in PGCs of F1 and F3 females

Among the male PGC genes with increased expression in female BaP-exposed E13.5 PGCs compared to vehicle controls were *Tdgf1*, the co-receptor of NODAL binding to its Type 2 Activin receptor, and *Lefty2*, whose expression is upregulated by NODAL signaling and which in turn inhibits NODAL signaling^44^*.* Both *Tdgf1* and *Lefty2* were in the top 25 DEGs when comparing female F1 2 mg/kg-day BaP (FDR *P* < 0.002), female F3 0.03 mg/kg-day BaP (FDR *P* < 0.04), and female F3 0.2 mg/kg-day BaP (FDR *P* < 0.001) to respective F1 and F3 controls, and *Tdgf1* was the top DEG when comparing female F3 2.0 mg/kg-day BaP to F3 vehicle controls (FDR *P* = 0.017). Consistent with these results, “Nodal signaling pathway” was the fourth most significant GO term in the female F2 2.0 mg/kg-day BaP females, and “positive regulation of activin receptor signaling pathway” was the tenth most significant GO term in the F1 0.2 mg/kg-day females, compared to respective controls.

**Figure 5:**
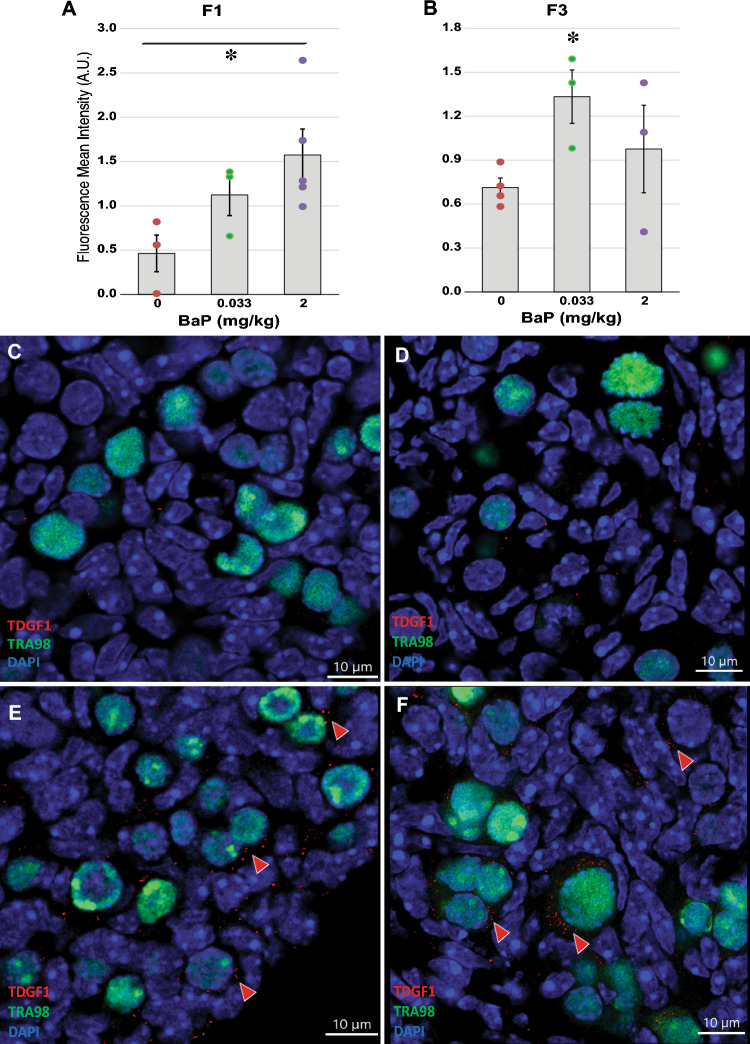
F0 dam BaP exposure increases protein expression of TDGF1 in ovaries of F1 and F3 female offspring. Pregnant OG2 females were exposed to BaP as for Figs. 1 and 2 and F1 and F3 ovaries were collected at E13.5 from female offspring and processed for immunofluorescence. Graphs show means ± SEM of mean fluorescence intensity in arbitrary units (AU). Dots represent values for individual embryos. F1 ovarian TDGF1 immunofluorescence increased with increasing F0 BaP dose (**A**, *P = 0.045, effect of BaP dose by linear regression; N = 3–5/group). F3 ovarian TDGF1 immunofluorescence was significantly increased in F3 ovaries descended from F0 dams treated with 0.033 mg/kg-day BaP compared to those descended from vehicle exposed F0 dams (**B**, *P = 0.034 Mann–Whitney test; N = 3–4/group). Representative images show cytoplasmic TDGF1 immunofluorescence in red in representative control F1 (**C**), control F3 (**D**), 2 mg/kg-day BaP F1 (**E**) and 0.033 mg/kg-day BaP F3 (**F**). Nuclei counterstained blue with DAPI and germ cell nuclei immunostained with TRA98 antibody (green). Arrowheads point to germ cells with positive cytoplasmic TDGF1 staining. Scale bars, 10 μm.

**Figure 6:**
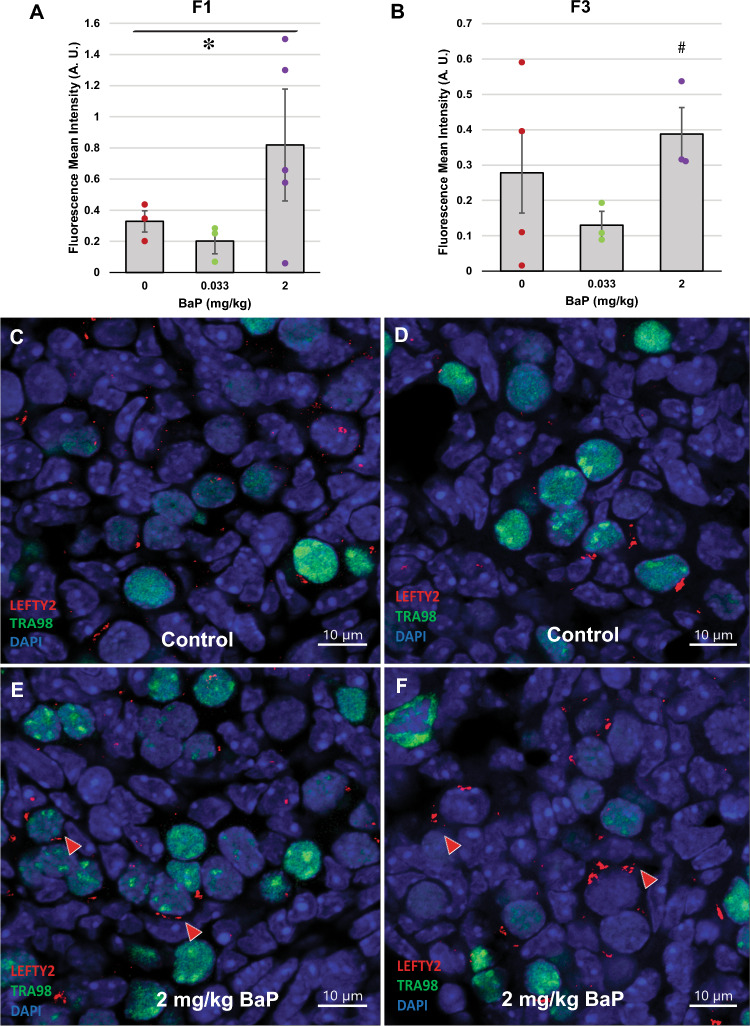
F0 dam BaP exposure increases protein expression of LEFTY2 in ovaries of F1 and F3 female offspring. Pregnant OG2 females were exposed to BaP as for Figs. 1 and 2 and F1 and F3 ovaries were collected at E13.5 from female offspring and processed for immunofluorescence. Graphs show means ± SEM of mean fluorescence intensity in arbitrary units (AU). Dots represent values for individual embryos. F1 ovarian LEFTY2 immunofluorescence increased with increasing F0 BaP dose (**A**, *P = 0.048, effect of BaP dose by linear regression; N = 3–5/group). F3 ovarian LEFTY2 immunofluorescence was non-significantly increased in F3 ovaries descended from F0 dams treated with 2 mg/kg-day BaP compared to those descended from 0.033 mg/kg-day exposed F0 dams (**B**, #*P* = 0.083, Mann–Whitney test; N = 3–4/group). Representative images show cytoplasmic LEFTY2 immunofluorescence in red in representative control F1 (**C**), control F3 (**D**), 2 mg/kg-day BaP F1 (**E**) and F3 (**F**). Nuclei counterstained blue with DAPI and germ cell nuclei immunostained with TRA98 antibody (green). Arrowheads point to cells with positive cytoplasmic LEFTY2 staining. Scale bars, 10 μm.

We performed immunofluorescence staining for TDGF1 and LEFTY2 and quantified expression using confocal microscopy and Imaris image analysis in F1 and F3 E13.5 vehicle and 0.033 and 2 mg/kg-day BaP ovaries. In the F1 ovaries, TDGF1 immunofluorescence increased with BaP dose in germ cell cytoplasm (*P* = 0.045, effect of BaP dose by linear regression; Fig. 5A). In the F3 ovaries, mean TDGF1 immunofluorescence was higher in both exposed groups than in controls, but the effect of BaP dose was not statistically significant by linear regression or non-parametric Kruskal Wallis test. Intergroup comparisons using non-parametric testing revealed that TDGF1 immunofluorescence was increased above control levels in the 0.033 mg/kg-day BaP-exposed group (*P* = 0.034, Mann Whitney test; Fig. 5B). In the F1 ovaries, LEFTY2 fluorescence increased with BaP dose (*P* = 0.048, effect of BaP dose by linear regression; Fig. 6A). In the F3 ovaries, mean LEFTY2 fluorescence in the 2 mg/kg-day group was non-significantly increased more than two-fold compared to the control group and more than threefold compared to the 0.033 mg/kg-day group (Fig. 6B), but the effect of BaP dose was not statistically significant by linear regression or non-parametric Kruskal Wallis test.

### Gestational F0 exposure affected gDNA methylation of F1 and F3 PGCs at several species of repetitive sequences

**Figure 7:**
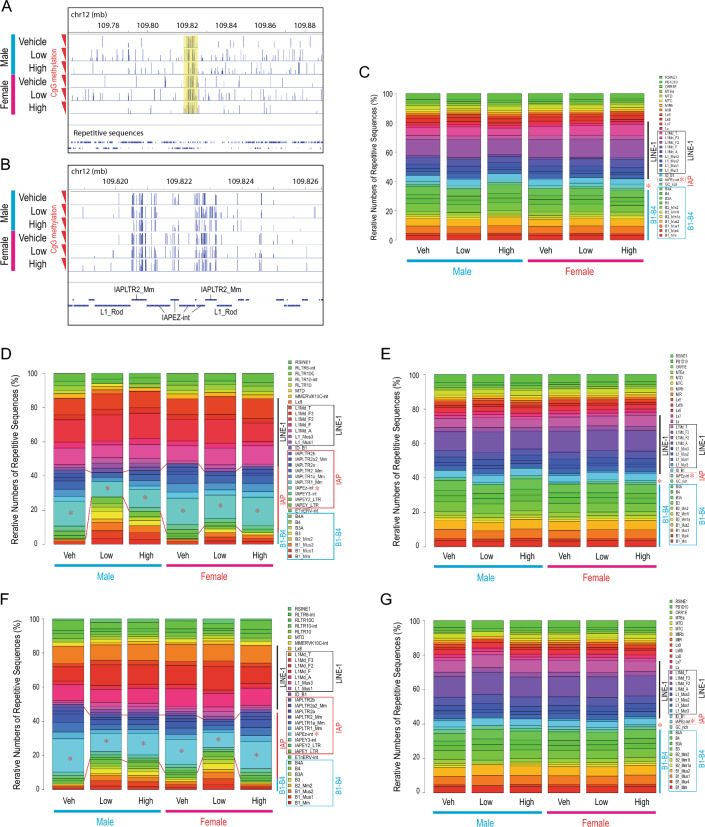
Effects of gestational F0 BaP exposure on genomic DNA methylation at repetitive sequences in F1 and F3 primordial germ cells. Genomic DNA (gDNA) of E13.5 PGCs isolated from F1 (**A**–**E**; N = 2/group) and F3 (**F**, **G**; N = 1/group) embryos descended from F0 females dosed with 0 (Veh), 0.2 (Low), or 2 (High) mg/kg-day BaP were subjected to whole genome bisulfite sequencing (WGBS). (**A**, **B**) Global gDNA demethylation except for IAP sequences. Panels A and B show CpG methylation profiles of a part of chromosome 12 in a 200-kb and 8-kb window, respectively. (**C**–**G**) Relative numbers of repetitive sequence copies in the whole mouse gDNA. Red asterisks indicate IAPEz-int sequence. Colored vertical bars and boxes indicate LINE-1, IAP, and B1-4 groups of repetitive sequences on the graphs and legends, respectively. (**C**) Top 35 repetitive sequences most frequently interrogated by WGBS. (**D**, **F**) Top 35 most strongly methylated repetitive sequences. Red lines between each column indicate the top and bottom boundaries of the IAP group repetitive sequences. (**E**, **G**) Top 35 unmethylated repetitive sequences.

We examined whether gestational exposure of F0 females affects the DNA methylome of E13.5 PGCs in F1 and F3 embryos by whole genome bisulfite sequencing (WGBS). gDNA of both male and female PGCs in E13.5 embryos was globally and strongly demethylated except for the IAP family of endogenous retroviruses, agreeing with previous studies^29^ (Fig. 7A,B). Neither sex of PGCs nor the F0 exposure caused significant changes in DNA methylation at IAP sequences.

To examine DNA methylation of the repetitive sequences in the mouse genome in more detail, we determined the genome-wide coverage of the RepeatMasker-registered repetitive sequences (GRCm38/mm10) by our WGBS. We identified the top 35 largest groups of repetitive sequences registered in the RepeatMasker file, 9 and 11 of which belonged to the LINE-1 family and B1-B4 repeats, respectively, whereas only one belonged to the IAP family (IAPEz-in indicated by asterisk) (Fig. 7C). In contrast, the top 35 most strongly methylated repetitive sequences (Fig. 7D,F) were greatly enriched with the IAP family (11 species) whereas 7 LINE-1 sequences were also methylated. Interestingly, whereas the B1-B4 repeats were minimally methylated in gDNA of PGCs after F0 exposure to vehicle, their methylation was augmented by exposure to BaP. The BaP-associated increase in B1-B4 repeats tended to be greater in F1 male PGCs than F1 female PGCs; instead, relative numbers of IAP sequences in the top 35 most methylated repetitive sequences decreased. In the F3 PGCs, the BaP-associated increase in B1-B4 repeats was similar in the 0.2 mg/kg-day BaP male and female PGCs (Fig. 7F). Detailed analyses focusing on F1 CpG methylation of IAPEz-int and B3 sequences by boxplots (Fig. S6A) and beeswarm plots (Fig. S6B) revealed disturbance in B3 methylation, including many hypermethylated B3 loci in the BaP-exposed PGCs (yellow band in Fig. S6B) whereas methylation of IAPEz-int was largely unaffected. The BaP-associated increase in B1–B4 repeats was reproduced with an independent set of WGBS data obtained from different animals (Fig. S6C). On the other hand, the profile of top 35 least methylated repetitive sequences (Fig. 7E,G) was almost identical to the WGBS coverage profile (Fig. 7C), agreeing with the robust gDNA demethylation in PGCs. The reason for or significance of the observed changes in the B1-B4 repeats and IAP sequences in the BaP-exposure E13.5 PGCs is unknown.

## Discussion

Our results demonstrate that in utero exposure via oral dosing of the PAH BaP to the pregnant F0 female mouse decreases the finite ovarian reserve in her directly exposed F1 daughters and in her unexposed F3 great grand-daughters. To the best of our knowledge, this is the first report of transgenerational effects on ovarian reserve by exposure to any PAH. Our E13.5 PGC RNA-seq results show that this is associated with statistically significant transgenerational upregulation in female PGCs of genes normally expressed highly in male PGCs and minimally expressed in female PGCs. In contrast, only modest effects were observed on sex-specific gene expression in male PGCs. BaP exposure did not affect the global DNA demethylation of gDNA that is expected in F1 E13.5 PGCs of both sexes. However, we observed increased methylation of B1-B4 repetitive sequences in BaP-exposed F1 and F3 offspring.

This is the first study to demonstrate multi- or trans-generational effects of a PAH at a human relevant dose, 0.033 mg/kg-day. BaP is a prototypical mutagenic PAH, which undergoes similar metabolism and has similar mechanisms of action as other mutagenic PAHs^1^. The daily mutagenic PAH intake of a highly, non-occupationally exposed woman is about 10 µg/day from smoking 40 cigarettes, 3 µg/day from breathing urban air, and 17 µg/day from eating grilled/smoked foods; or a total of 0.5 µg/kg-day for a 60 kg woman, for a cumulative dose over 39 weeks of pregnancy of 137 µg/kg^1–3,45^. 0.033 mg/kg-day is about tenfold higher than the average daily dose of mutagenic PAHs of a highly exposed woman after allometric interspecies dose correction^46^. With our 10-day gestational dosing window, the cumulative dose of 0.33 mg/kg is about one third of the estimated cumulative dose during pregnancy of a highly exposed woman after allometric interspecies correction. Remarkably, this dose resulted in 50% fewer germ cells at E13.5 and primordial follicles at PND21 in the F3 female offspring. Prior studies have shown distribution of BaP and its metabolites to ovaries and embryos after oral and inhalational dosing. Oral dosing of pregnant female mice from E6.5–11.5 with two of the doses used in the present paper, 0.2 and 2 mg/kg-day, resulted in total BaP metabolite concentrations in the whole female E13.5 embryos of 1.9 and 2.6 ng/g tissue, respectively; due to tissue requirements, it was not possible to measure BaP metabolites in embryonic ovaries^20^. In contrast, metabolite concentrations in the placentas were about half those in the embryos^20^. The results were consistent with efficient transfer of BaP and its metabolites via the placenta and embryonic metabolism of BaP^20^. BaP metabolites were detectable in the ovaries of adult F344 rats as many as 28 days after a single 5 mg/kg oral dose, while the parent compound was detectable at 7 days, and was no longer detectable at 14 or more days after dosing^47^. In female F344 rats exposed to 3 doses of BaP via inhalation for 4 h/day for 14 days, BaP metabolite concentrations in ovaries in the highest dose group (100 μg/m^3^) were about ninefold higher than lung and about threefold higher than liver^48^.

In mice and humans, germ cells in the developing ovaries enter meiosis and arrest in the diplotene stage of the first meiotic prophase prior to birth^49^. Therefore, unlike males, who have germline stem cells present in the testes throughout life, females are born with a finite number of germ cells, called oocytes, which are packaged into primordial follicles during the first postnatal week in mice^49^ and by the end of the second trimester in humans^49,50^. Depletion of the ovarian reserve of primordial follicles results in premature cessation of ovarian function. When this occurs in women prior to the age of 40, it is called premature ovarian insufficiency (POI), and it is a major cause of infertility and impaired fecundity in women^51–53^. POI is associated with increased risk of multiple adverse health outcomes. Women with POI have increased risk of dyslipidemia, endothelial dysfunction, thromboembolism, hypertension, metabolic syndrome, coronary artery disease, and stroke^54,55^. Women with POI also have increased risk of cognitive decline and Alzheimer’s disease^56–58^, and osteoporosis^54,56,59,60^. While it has been known for some time from experimental studies that preconceptional and gestational exposure to BaP and other PAHs depletes the ovarian reserve of primordial follicles and causes infertility in the F1 female offspring^12,14,18,61^, the data presented herein support that ancestral exposure to PAHs may be a heretofore unappreciated cause of POI. This is significant not just for fertility, but also for the overall health of women, given the adverse effects of POI on cardiovascular, brain, and bone health^20,47^.

The transgenerational depletion of the ovarian reserve caused by F0 BaP exposure was associated with statistically significant transgenerational increases in expression of male PGC marker genes in the female E13.5 PGC transcriptomes. Two male PGC marker genes, *Tdgf1* and *Lefty2,* which are components of the Nodal signaling pathway, were significantly upregulated in female PGCs across generations and BaP dose groups. Further supporting that BaP transgenerationally upregulates Nodal signaling in female germ cells, Nodal receptor genes, *Acvr1b,* and *Acvr2*^62^, were also upregulated in several female BaP-exposed groups. Nodal signaling prevents male PGCs from entering meiosis during the developmental stage when female germ cells are beginning to enter meiosis^62–64^. GO analysis identified GO terms related to meiosis that were affected by F0 BaP exposure across generations and doses in females. Among the genes associated with the meiosis GO terms were *Meiob*, *Syce1 and 3*, *Sycp2* and *3*, *Tex11, Tex12, Tex15,* and *Ccnb3*, which were down-regulated in F1 2 mg/kg-day and F3 0.2 mg/kg-day females compared to respective controls. All of these, except *Meiob* and *Ccnb3*, are synaptonemal complex components^65^. *Ccnb3* is required for the initiation of anaphase I in oocyte meiosis^66^. *Meiob* is a single strand DNA binding protein, which is required for meiotic recombination^67^. Taken together these results suggest that meiosis initiation in female germ cells directly or ancestrally BaP exposed may be disturbed and that this may play a role in the transgenerational ovarian phenotype. Future studies should undertake detailed analyses of meiosis I progression in embryonic ovaries directly or ancestrally exposed to BaP.

Analyses of DEGs that differed by at least 1.5-fold and had unadjusted *p*-values < 0.05 between BaP-exposed and respective control groups revealed many GO terms related to immune responses and inflammation. Among the genes driving these GO terms were multiple c–c motif chemokines (*Ccl2, Ccl3, Ccl6, Ccl7, Ccl12, Ccl17, Ccl25, Ccl28*) and to a lesser extent their G protein-coupled receptors (*Ccr7, Ccr12*), which were increased across multiple BaP dose groups and generations. We previously reported increased expression of *Ccl2* and *Ccl7* by RNAseq of whole F1 E13.5 ovaries exposed to BaP from E6.5–11.5, confirmed with immunofluorescence for CCL2^20^. CCL2, CCL3, CCL7, and CCL12 are monocyte chemoattractants^68^, which contribute to the tumor microenvironment of ovarian cancers^69–71^. *Ccl2* expression was reportedly upregulated in cultured Type II alveolar pneumocytes by another PAH, 1-methylanthracene^72^. In the neonatal rat ovary inflammation induced by lipopolysaccharide administration caused ovarian reserve depletion^73^. Our results suggest that F0 BaP exposure may cause inflammation in the developing ovaries across generations.

In our second experiment, we tested the relative sensitivity to ovarian follicle depletion of two different ovarian developmental windows, the PGC proliferative E6.5–11.5 window and the predominantly meiotic E12.5–17.5 window. We previously published that oral dosing with 2 mg/kg-day BaP of C57BL/6J dams during either window significantly reduced numbers of ovarian primordial, primary and secondary follicles at puberty in the F1 female offspring, with no effect of exposure window^36^. The F2 and F3 mice for Experiment 2 in the present paper were descended from the siblings of those F1 females. Interestingly, decreased ovarian reserve was not observed in the F2 and F3 females in this experiment. We think that the most likely reason is the shorter dosing windows and that exposure during both developmental windows is necessary for the transgenerational effect on ovarian reserve. The mice for Experiment 1 were transgenic OG2 mice on a C57BL/6J genetic background, while those for Experiment 2 were wild type C57BL/6J mice. However, we think it is unlikely that this genetic difference is the reason for the lack of transgenerational effects. Using mice heterozygous for the Glutamate Cysteine Ligase Modifier subunit, *Gclm*+*/−* mice, which are also on a C57BL/6J genetic background, we found that follicles per ovary were significantly decreased in F2 *Gclm*+*/−* mice descended from F0 mothers dosed for the longer, E6.5–15.5 dosing window with 2 mg/kg-day BaP (Fig. S1), supporting that the longer dosing window is required to decrease ovarian reserve in F2 and F3 females.

In conclusion, our results demonstrate that exposure of pregnant F0 females to the PAH BaP greatly diminishes the size of the ovarian germ cell pool in their descendants in a transgenerational manner. Decreased size of the ovarian germ cell pool inevitably results in premature ovarian follicle depletion and early ovarian senescence due to the non-renewable nature of the mammalian female germ cell pool. Our experimental paradigm did not allow us to distinguish whether this phenotype is transmitted via the female or male germline, and future studies should be conducted to delineate whether one or both germlines transmit the phenotype. Our results further demonstrate that F0 BaP exposure results in transgenerational masculinization of the female E13.5 germ cell transcriptomes, which we postulate plays a role in mediating the diminished ovarian reserve phenotype. Our results point to possible roles for inflammation and activation of Nodal signaling as potential mechanisms by which F0 exposure to BaP causes transgenerational depletion of the ovarian reserve.

## Methods

### Animals

Mice with germ cell-specific expression of EGFP under control of a *Pou5f1* (also called *Oct4*) promoter and distal enhancer (B6;CBA-Tg(Pou5f1-EGFP)2Mnn/J; referred to as OG2 mice^35^) were originally purchased from Jackson Labs (Strain #004654) and have been backcrossed 9 times onto a C57BL/6J genetic background and maintained as heterozygotes in our colony. OG2 mice were used for Experiment 1. Wild type C57BL/6J mice (Jackson Labs, Strain #000664) were used for Experiment 2. Mice deficient in *Gclm* and littermate controls on a C57BL6/J background were used for Experiment 3^74^. Mice were housed in an American Association for the Accreditation of Laboratory Animal Care International-accredited facility at the University of California Irvine (UC Irvine), with free access to deionized water and soy-free laboratory chow (Harlan Teklad, 2919), on a 14:10 h light–dark cycle. Temperature was maintained at 21–23 °C. The experimental protocols were carried out in accordance with the Guide for the Care and Use of Laboratory Animals^75^ and ARRIVE Guidelines^76^ and were approved by the Institutional Animal Care and Use Committee at UC Irvine.

### Experimental design

*Experiment 1, Transgenerational effects of *in utero* BaP on gonadal germ cell numbers and PGC transcriptomes and methylomes:* Homozygous 10–14 weeks old male and female OG2 mice were mated on the evening of the female’s proestrus, determined by daily vaginal cytology. The next morning females with vaginal plugs were considered to be pregnant and that time point was designated embryonic day (E) 0.5. Pregnant F0 dams were fed sesame oil containing 0, 0.033, 0.2, or 2 mg/kg-day BaP (Sigma Aldrich, #48564) by oral pipetting into the cheek pouch daily from E6.5–11.5 for collection of PGCs or whole gonads from E13.5 F1 embryos or from E6.5–15.5 for generation of F2 and F3 offspring. We previously estimated that a highly environmentally exposed woman is exposed to 0.5 µg/kg-day of mutagenic PAHs, including BaP, for a cumulative dose of 1.0 mg over the course of a 39 week pregnancy, which is equivalent to 3.7 µg/kg-day in mice after allometric interspecies correction^1–3,20,45,77^. The 0.033 mg/kg-day dose in the present study provides a cumulative dose of 0.33 mg over 10 days, which is about one third of the estimated cumulative dose of mutagenic PAHs during pregnancy of a highly environmentally exposed woman. F1 and F2 non-littermate males and females from the same F0 dose group were mated to generate F2 and F3 generations, respectively. F1, F2, and F3 females were euthanized at PND21 for ovarian follicle enumeration. Pregnant F0, F1, and F2 dams were euthanized by CO_2_ asphyxiation on E13.5 for F1, F2, and F3 embryonic gonad collection and PGC isolation.

*Experiment 2, Transgenerational effects of BaP exposure during mitotic or meiotic ovarian developmental window:* C57BL/6J male and female mice were mated on the evening of the female’s proestrus as described above, and pregnant females were orally dosed with 2 mg/kg-day BaP or oil vehicle from E6.5–11.5 (germ cell mitotic window during ovarian development) or from E12.5–17.5 (germ cell meiotic window of ovarian development). F0 dams were allowed to give birth to the F1 generation. To produce subsequent generations, at least one F1 female from each litter was mated with a non-littermate F1 male of the same treatment group to yield the F2 generation. F2 females were mated with non-littermate F2 males of the same treatment group to produce the F3 generation. F1 and F3 females were euthanized on the day of first vaginal estrus (puberty onset^78^), determined by vaginal cytology, and F2 females were euthanized at 4 to 5 months of age for ovarian follicle counts. Follicle count data from pubertal F1 females have already been reported ^36^.

*Experiment 3: Effects of F0 BaP exposure on ovarian follicle numbers in F2 offspring:* Pregnant F0 female *Gclm*+*/−* mice, which had been mated with *Gclm*+*/−* male mice, were dosed with 0 or 2 mg/kg-day from E6.5–15.5, and F1 offspring were mated as for Experiment 1 to generate F2 female offspring. *Gclm+/-* F2 female offspring were euthanized at 4–5 months of age for ovarian follicle enumeration.

### Germ cell and follicle enumeration

Randomly selected E13.5 ovaries and testes from F1-F3 embryos and ovaries from post-natal females were harvested and immediately fixed in Bouin’s solution at 4 °C for 3 h, washed in 50% ethanol three times, and stored in 70% ethanol until embedding in glycolmethacrylate resin (Technovit 8100; Heraeus Kulzer GmBH, Wehrheim, Germany). Embedded E13.5 gonads and postnatal ovaries were sectioned at 20 µm and stained with hematoxylin and eosin. Stereological methods were used to obtain unbiased estimates of germ cell (E13.5) or ovarian follicle (PND21) numbers^20,79^. E13.5 germ cells were identified based on their morphology (Fig. S7). Germ cells and ovarian follicles were counted blind to treatment group using Stereo Investigator software (MBF Bioscience) with an Olympus BX40 light microscope equipped with 4 × UPlanFl, 10 × Plan, 40 × UApo N340, and 60 × PlanApo objectives, a joystick controller for a motorized XY stage (Ludl Electronic Products), and an Optronics MicroFire digital camera. The fractionator/optical dissector method was used to obtain unbiased and efficient estimates of germ cell or follicle numbers by counting germ cells or follicles in a defined fraction of the whole gonad or PND 21 ovary^79^. For E13.5 gonads, every second section was counted using 5625 μm^2^ (75 μm × 75 μm) counting frames that were superimposed onto the sections in sampling grids subdivided into 15,625 μm^2^ (125 μm × 125 μm) squares. Germ cells were counted only if the germ cell fell within the counting frame and/or touched the inclusion boundaries and did not touch the exclusion boundaries. Lastly, the optical dissector height was set to 10 μm with guard zones on the top and the bottom of the section to account for irregularities of the sections. By multiplying the raw counts by the reciprocals of the counting fractions, the number of germ cells in the entire gonad was estimated. For PND 21 and pubertal ovaries, primordial, primary, and secondary follicle numbers were similarly counted. Three levels of sampling were used to determine the estimated number of follicles in the ovary- every 3rd section of the ovary with 10,000 μm^2^ (100 μm × 100 μm) counting frames and 30,625 μm^2^ (175 μm × 175 μm) sampling grids. Follicles were counted if the oocyte fell within the counting frame and/or touched the inclusion boundaries within the middle 10 μm of the sections. Antral follicles were followed through every section to avoid double counting using an Olympus BX-60 light microscope. Follicles were classified as primordial (a single layer of flattened granulosa cells with no more than one cuboidal granulosa cell), primary (a single layer with two or more cuboidal granulosa cells), secondary (more than one layer of granulosa cells with no antrum), or antral (multiple layers of granulosa cells with antrum) follicles. Atretic follicles were identified as previously described^14,80,81^.

### Isolation of mouse PGCs

E13.5 embryos were collected from timed-mated OG2 female mice, and their gonads with attached mesonephros were isolated by dissection under a microscope. Sex of the embryonic gonads were determined by morphological characteristics^20^, and the gonads were subjected to digestion using Embryoid Body Dissociation Kit (Miltenyi Biotec, #130-096-348) at 37 °C to obtain single cell suspensions. Enzymatic and physical dissociation was stopped by adding DMEM medium supplemented with 10% fetal calf serum. PGCs expressing EGFP from the germline-specific *Pou5f1* promoter were enriched by FACS as we previously described^39^. Briefly, the resuspended cells were filtered through a 35 µm nylon mesh (Coming, #352232) and supplemented with propidium iodide (2 µg/ml) to remove dead cells. We first excluded cell debris and cell clumps (P1, SSC-A/FSC-A and P2, FSC-H/FSC-A), then gated out dead cells using propidium iodide (P3, PI-A/FSC-A; Fig. S2). Finally, we isolated EGFP-positive germ cells and excluded EGFP negative somatic cells (P4, GFP-A/FSC-A; Fig. S2). All sorting was performed on a BD FACSAria II cell sorter (BD Biosciences) equipped with four lasers (405 nm, 488 nm, 561 nm, and 640 nm). For downstream assays (RNA-Seq or WGBS), 2000–10,000 PGCs were sorted into a tube containing 500 μl of 1% BSA and centrifuged at 300 g for 8 min. The supernatant was carefully removed, and the cell pellets were frozen on dry ice and kept at − 80 °C until used.

### RNA-seq

cDNA was synthesized directly from 20,000 to 30,000 F1, F2, and F3 FACS-enriched PGCs using SMARTer Ultra Low Input RNA Kit for Sequencing (TAKARA Bio, San Jose, USA) using the oligo(dT) primer provided in the kit, and Illumina sequencing libraries were generated using Low Input DNA Library Prep Kit (TAKARA Bio). Libraries were sequenced using an Illumina NextSeq 500 deep sequencer with high output flowcells to obtain 75 + 75 nt paired-end FASTQ reads, which were subjected to quality control analysis using the fastQC tool (Babraham Institute, Cambridge, UK). After adaptor sequences and low-quality reads (< 30) were removed using the Trim Galore! tool, FASTQ reads were aligned to the GRCm38/mm10 mouse reference genome using the STAR aligner software^82^ to obtain BAM files. At least 16 million uniquely mapped leads were obtained for each library.

To evaluate expression of mRNA transcripts, aligned reads in the BAM format were assigned to exons of the mm10 gene model and counted using the Bioconductor package Rsubread^83^. The mRNA expression counts were normalized using the negative binominal trimmed mean of M-values (TMM) method implemented by the Bioconductor package edgeR^84^. The normalized counts were subjected to unsupervised hierarchical clustering analysis and visualization using Cluster^85^ and Java TreeView^86^. Differentially expressed genes (DEGs) were identified using the generalized linear model likelihood ratio test implemented by edgeR^84^. For determination of sex-specific DEGs, male and female PGCs isolated from unexposed with a set of criterion of false discovery rate (FDR) < 0.05 and greater than twofold changes. Exposure-specific DEGs were sorted by statistical significance (most significant genes were ranked at the top).

### Whole genome bisulfite sequencing

gDNA was isolated from the FACS-enriched F1 and F3 PGCs using AllPrep Micro Kit and subjected to synthesis of bisulfite-converted Illumina sequencing libraries using EZ DNA Methylation-Gold kit (Zymo Research, Irvine, USA) and Accel-NGS® Methyl-Seq DNA Library Kit (Swift Biosciences/IDT, Coralville, USA). Bisulfite conversion efficiency was evaluated as we previously described^87^ and confirmed > 99%. Libraries were sequenced using an Illumina NovaSeq 6000 deep sequencer with S4 flowcells to obtain 150 + 150 nt paired-end FASTQ reads, which were subjected to quality control analysis using fastQC. After adaptor sequences and low-quality reads (< 30) were removed using the Trim Galore!, FASTQ reads were analyzed using the Bismark pipeline as we previously described to obtain genome-wide profiles of CpG methylation^88^. Approximately 100 million uniquely mapped leads were obtained for each library. RepeatMasker-estimated coordinates of repetitive sequences in the GRCm38/mm10 mouse reference genome were downloaded from the UCSC browser, and methylation profiles of each repetitive sequences were determined by R scripts.

### Immunofluorescence

E13.5 gonads were fixed in 4% PFA for 2–3 h at 4 °C and cryoprotected in 30% sucrose prior to embedding in OCT and freezing. The frozen gonads were cryosectioned at 7 μm thickness. Sections were pretreated in 10 mM citric acid for 15 min at 95 °C, blocked in PBS-Triton X-100 solution with 5% normal goat serum and 1% BSA for 1 h and incubated with primary antibodies, rabbit anti-TDGF1 (1: 200; Cell Signaling #2818), rabbit anti-LEFTY2 (1:100; Abcam ab22569), and rat anti-TRA98 (1:200; Abcam ab82527), at 4 °C overnight. After washing, the primary antibodies were detected with corresponding secondary antibodies, goat anti-rabbit Alexa Fluor 633 (ThermoFisher, A21070) and goat anti-rat Alexa Fluor 488 (ThermoFisher, A11006), for 1 h at room temperature. Sections were treated with autofluorescence quenching reagent (ThermoFisher, R37630) and stained with DAPI for nuclei staining. Slides were mounted in Prolong glass antifade mountant (ThermoFisher, P36980). Images of fluorescently TRA98-labeled germ cells with TDGF1 or LEFTY2 were generated using Zeiss LSM 900 Airyscan 2 microscope and processed using the Bitplane Imaris imaging software (v.9.9.0). Image J was used to quantify mean fluorescence intensity of TDGF1 or LEFTY2 immunopositive cells in a whole ovary section by an investigator blind to experimental group. Briefly, images were converted to 16-bit grayscale and the whole ovary was selected using “Polygon” tool. Then, the mean intensity of fluorescence signal was measured and used for data analysis.

### Statistics

Data are presented as mean ± SEM unless otherwise noted. For germ cell and ovarian follicle counts, Generalized Estimating Equations with exchangeable correlation matrices were used to adjust for correlation among counts in females from the same litter or, for F2 and F3 females, descended from the same F0 dam. For immunofluorescence quantitation, linear regression and Kruskal Wallis test were used to examine the effects of BaP dose. For comparisons of two means, t-tests or non-parametric Mann Whitney tests were used, as appropriate. Statistical significance was set to *P* < 0.05. Analyses were carried out using SPSS 28 for Mac or R.

Permutation tests were performed to evaluate the likelihood of randomly selecting sex-specific DEGs (Table S1) out of all genes whose maximum expression count exceeds 100 (Table S2). A set of genes was randomly sampled from the list of all expressed genes (Table S2) with varying sample sizes (50, 100, 200) and then compared to the list of sex-specific DEGs (Table S1) to calculate a fraction by dividing the number of sex-specific DEGs by the sample size. This process was iterated 5,000 times to draw a histogram of the 5,000 fraction data, and the distribution of the fraction data was fitted to the γ-distribution with the moment matching estimation method using the R function fitdist.

## Supplementary Information

Supplementary Figures.

Supplementary Table S1.

Supplementary Table S2.

Supplementary Table S3.

Supplementary Table S4.

Supplementary Table S5.

Supplementary Table S6.

Supplementary Table S7.

Supplementary Table S8.

Supplementary Table S9.

Supplementary Table S10.

Supplementary Table S11.

## Data Availability

RNA-sequencing and Whole Genome Bisulfite Sequencing data that support the findings of this study have been deposited in GEO: RNA-seq, GSE213812. WGBS (both main and supplemental data), GSE213947 and GSE230356. Additional data supporting the findings of this study are available within the paper, in its supplementary information files, or from the corresponding author upon request.

## References

[1] ATSDR. *Toxicological Profile for Polycyclic Aromatic Hydrocarbons* (US Department of Health and Human Services, Public Health Service, Agency for Toxic Substances and Disease Registry, 1995). 38091452

[2] Menzie CA, Potocki BB, Santodonato J (1992). Ambient concentrations and exposure to carcinogenic PAHs in the environment. Environ. Sci. Technol..

[3] Lodovici M, Akpan V, Evangelisti C, Dolara P (2004). Sidestream tobacco smoke as the main predictor of exposure to polycyclic aromatic hydrocarbons. J. Appl. Toxicol..

[4] NHANES. Fourth National Report on Human Exposure to Environmental Chemicals, Updated Tables, January 2019 (Department of Health and Human Services, Centers for Disease Control and Prevention, 2019).

[5] Xue W, Warshawsky D (2005). Metabolic activation of polycyclic aromatic hydrocarbon and heterocyclic aromatic hydrocarbons and DNA damage: A review. Toxicol. Appl. Pharmacol..

[6] Burczynski ME, Penning TM (2000). Genotoxic polycyclic aromatic hydrocarbon *ortho*-quinones generated by aldo–keto reductases induce CYP1A1 via nuclear translocation of the aryl hydrocarbon receptor. Cancer Res..

[7] Penning TM (2004). Aldo–keto reductases and formation of polycyclic aromatic hydrocarbon *o*-quinones. Methods Enzymol..

[8] Penning TM, Ohnishi ST, Ohnishi T, Harvey RG (1996). Generation of reactive oxygen species during the enzymatic oxidation of polycyclic aromatic hydrocarbon trans-dihydrodiols catalyzed by dihydrodiol dehydrogenase. Chem. Res. Toxicol..

[9] Cavalieri EL, Rogan EG (1995). Central role of radical cations in metabolic activation of polycyclic aromatic hydrocarbons. Xenobiotica.

[10] van Lipzig MMH, Vermeulen NPE, Gusinu R, Legler J, Frank H, Seidel A, Meerman JHN (2005). Formation of estrogenic metabolites of benzo[a]pyrene and chrysene by cytochrome P450 activity and their combined and supra-maximal estrogenic activity. Environ. Toxicol. Pharmacol..

[11] Plíšková M, Vondráček J, Vojtěšek B, Kozubík A, Machala M (2005). Deregulation of cell proliferation by polycyclic aromatic hydrocarbons in human breast carcinoma MCF-7 cells reflects both genotoxic and nongenotoxic events. Toxicol. Sci..

[12] MacKenzie KM, Angevine DM (1981). Infertility in mice exposed in utero to benzo(a)pyrene. Biol. Reprod..

[13] Lundgaard Riis M, Jørgensen A (2022). Deciphering sex-specific differentiation of human fetal gonads: Insight from experimental models. Front. Cell Dev. Biol..

[14] Lim J, Lawson GW, Nakamura BN, Ortiz L, Hur JA, Kavanagh TJ, Luderer U (2013). Glutathione-deficient mice have increased sensitivity to transplacental benzo[a]pyrene-induced premature ovarian failure and ovarian tumorigenesis. Cancer Res..

[15] Nakamura BN, Mohar I, Lawson GW, Hoang YD, Cortés MM, Ortiz L, Patel R, Rau BR, McConnachie L, Kavanagh TJ, Luderer U (2012). Increased sensitivity to testicular toxicity of transplacental benzo[a]pyrene exposure in male glutamate cysteine ligase modifier subunit *Gclm−/−*) knockout mice. Toxicol. Sci..

[16] Lim J, Kong W, Lu M, Luderer U (2016). The mouse fetal ovary has greater sensitivity than the fetal testis to benzo[a]pyrene-induced germ cell death. Toxicol. Sci..

[17] Lim J, Luderer U (2018). Glutathione deficiency sensitizes cultured embryonic mouse ovaries to benzo[*a*]pyrene-induced germ cell apoptosis. Toxicol. Appl. Pharmacol..

[18] Matikainen T, Moriyama T, Morita Y, Perez GI, Korsmayer SJ, Sherr DH, Tilly JL (2002). Ligand activation of the fetal aromatic hydrocarbon receptor transcription factor drives Bax-dependent apoptosis in developing fetal ovarian germ cells. Endocrinology.

[19] Matikainen T, Perez GI, Jurisicova A, Pru JK, Schlezinger JJ, Ryu H-Y, Laine J, Sakai T, Korsmeyer SJ, Casper RF, Sherr DH, Tillly JL (2001). Aromatic hydrocarbon receptor-driven Bax gene expression is required for premature ovarian failure caused by biohazardous environmental chemicals. Nat. Genet..

[20] Lim J, Ramesh A, Shioda T, Leon Parada K, Luderer U (2022). Sex differences in embryonic Gondal transcriptomes and benzop[a]pyrene metabolite levels after transplacental exposure. Endocrinology.

[21] Rissman EF, Adli M (2014). Minireview: Transgenerational epigentic inheritance: Focus on endocrine disrupting compounds. Endocrinology.

[22] Kovalchuk I (2012). Transgenerational epigenetic inheritance in animals. Front. Gen..

[23] Xavier MJ, Roman SD, Aitken RJ, Nixon B (2019). Transgenerational inheritance: How impacts to the epigenetic and genetic information of parents affect offspring health. Hum. Reprod. Update.

[24] Nilsson EE, Ben Maamar M, Skinner MK (2022). Role of epigenetic transgenerational inheritance in generational toxicology. Environ. Epigenet..

[25] Janesick AS, Shioda T, Blumberg B (2014). Transgenerational inheritance of prenatal obesogen exposure. Mol. Cell. Endocrinol..

[26] Mohamed E-SA, Song W-S, Oh S-A, Park Y-J, You Y-A, Lee S, Choi J-Y, Kim Y-J, Jo I, Pang M-G (2010). The transgenerational impact of benzo[a]pyrene on murine male fertility. Hum. Reprod..

[27] Magnúsdóttir E, Surani MA (2014). How to make a primordial germ cell. Development.

[28] de Sousa Lopes SM, Hayashi K, Surani MA (2007). Proximal visceral endoderm and extraembryonic ectoderm regulate the formation of primordial germ cell precursors. BMC Dev. Biol..

[29] Seisenberger S, Andrews S, Krueger F, Arand J, Walter J, Santos F, Popp C, Thienpoint B, Dean W, Reik W (2012). The dynamics of genome-wide DNA methylation reprogramming in mouse primordial germ cells. Mol. Cell.

[30] Guo F (2015). The transcriptome and DNA methylome landscapes of human primordial germ cells. Cell.

[31] Kobayashi H, Sakurai T, Miura F, Imai M, Mochiduki K, Yanagisawa E, Sakashita A, Wakai T, Suzuki Y, Ito T, Matsui Y, Kono T (2013). High-resolution DNA methylome analysis of primordial germ cells identifies gender-specific reprogramming in mice. Genome Res..

[32] Skinner MK, Guerrero-Bosagna C, Haque MM (2015). Environmentally induced epigenetic transgenerational inheritance of sperm epimutations promote genetic mutations. Epigenetics.

[33] Skinner MK, Guerrero-Bosagna C, Haque M, Nilsson E, Bhandari R, McCarrey JR (2013). Environmentally induced transgenerational epigenetic reprogramming of primordial germ cells and the subsequent germ line. PLoS ONE.

[34] Pesce M, Wang X, Wolgemuth DJ, Schöler H (1998). Differential expression of the Oct-4 transcription factor during mouse germ cell differentiation. Mech. Dev..

[35] Szabó P, Hübner K, Schöler H, Mann JR (2002). Allele-specific expression of imprinted genes in mouse migratory primordial germ cells. Mech. Dev..

[36] Malott KF, Leon Parada K, Lee M, Swanson E, Luderer U (2022). Gestational benzo[a]pyrene exposure destroys F1 ovarian germ cells through mitochondrial apoptosis pathway and diminishes surviving oocyte quality. Toxicol. Sci..

[37] Richardson BE, Lehmann R (2010). Mechanisms guiding primordial germ cell migration: Strategies from different organisms. Nat Rev Molec Cell Biol.

[38] Culty M (2013). Gonocytes, from the fifties to the present: Is there a reason to change the name?. Biol. Reprod..

[39] Shioda K, Odajima J, Blumberg B, Shioda T (2022). Transgenerational transcriptomic and DNA methylome profiling of mouse fetal testicular germline and somatic cells after exposure of pregnant mothers to tributyltin, a potent obesogen. Metabolites.

[40] Saitou M, Yamaji M (2012). Primordial germ cells in mice. Cold Spring Harb. Perspect. Biol..

[41] Sakashita A, Kawabata Y, Jincho Y, Tajima S, Kumamoto A, Kobayashi H, Matsui Y, Kono T (2015). Sex specification and heterogeneity of primordial germ cells in mice. PLoS ONE.

[42] Eden E, Navon R, Steinfeld I, Lipson D, Yakhini Z (2009). GOrilla: A tool for discovery and visualization of enriched GO terms in ranked gene lists. BMC Bioinformatics.

[43] Supek F, Bošnjak M, Škunca N, Šmuc T (2011). REVIGO summarizes and visualizes long lists of gene ontology terms. PLoS ONE.

[44] Harpelunde Poulsen K, Jørgensen A (2019). Role of Nodal signalling in testis development and initiation of testicular cancer. Reproduction.

[45] Shopland, D. R., Burns, D. M., Benowitz, N. L. & Amacher, R. H. in *Smoking and Tobacco Control Monographs* Vol. 13 1–236 (U.S. Department of Health and Human Services, Public Health Service, National Institutes of Health, National Cancer Institute, Washington, D.C., 2001).

[46] USEPA. *Recommended Use of Body Weight3/4 as the Default Method in Derivation of the Oral Reference Dose*. (ed Office of the Scientific Advisor Risk Assessment Forum) (U.S. Environmental Protection Agency, Washington, DC, 2011). EPA/100/R11/0001.

[47] Ramesh A, Archibong AE, Niaz MS (2010). Ovarian susceptibility to benzo[a]pyrene: Tissue burden of metabolites and DNA adducts in F-344 rats. J. Toxicol. Environ. Health Part A.

[48] Archibong AE, Ramesh A, Inyang F, Niaz MS, Hood DB, Kopsombut P (2012). Endocrine disruptive actions of inhaled benzo[a]pyrene on ovarian function and fetal survival in fisher F344 adult rats. Reprod. Toxicol..

[49] Pepling ME (2006). From primordial germ cell to primordial follicle: Mammalian female germ cell development. Genesis.

[50] Oktem O, Oktay K (2008). The ovary. Anatomy and function throughout human life. Ann. N. Y. Acad. Sci..

[51] van Noord PAH, Boersma H, Dubas JS, te Velde ER, Dorland M (1997). Age at natural menopause in a population-based screening cohort: The role of menarche, fecundity, and lifestyle factors. Fertil. Steril..

[52] Greene AD, Patounakis G, Segars JH (2014). Genetic associations with diminished ovarian reserve: A systematic review of the literature. J. Assist. Reprod. Genet..

[53] Panay N, Anderson RA, Nappi RE, Vincent AJ, Vujovic S, Webber L, Wolfman W (2020). Premature ovarian insufficiency: An international menopause society white paper. Climacteric.

[54] Dubey RK, Imthurn B, Barton M, Jackson EK (2005). Vascular consequences of menopause and hormone therapy: Importance of timing of treatment and type of estrogen. Cardiovasc. Res..

[55] Stevenson JC, Collins P, Hamoda H, Lambrinoudaki I, Maas A, Maclaran K, Panay N (2021). Cardiometabolic health in premature ovarian insufficiency. Climacteric.

[56] Silva I, Mor G, Naftolin F (2001). Estrogen and the aging brain. Maturitas.

[57] Pines A (2014). Surgical menopause and cognitive decline. Climacteric.

[58] Bove R, Secor E, Chibnik LB, Barnes LL, Schneider JA, Bennett DA, De Jager PL (2014). Age at surgical menopause influences cognitive decline and Alzheimer pathology in older women. Neurology.

[59] Svejme O, Ahlborg HG, Nilsson J-Å, Karlsson MK (2012). Early menopause and risk of osteoporosis, fracture and mortality: A 34-year prospective observational study in 390 women. BJOG.

[60] Shuster LT, Gostout BS, Grossardt BR, Rocca WA (2008). Prophylactic oophorectomy in premenopausal women and long-term health. Menopause Int..

[61] Jurisicova A, Taniuchi A, Li H, Shang Y, Antenos M, Detmar J, Xu J, Matikainen T, Hernandez AB, Nunez G, Casper RF (2007). Maternal exposure to polycyclic aromatic hydrocarbons diminishes murine ovarian reserve via induction of *Harakiri*. J. Clin. Invest..

[62] Wu Q, Kanata K, Saba R, Deng C-X, Hamada H, Saga Y (2013). Nodal/activin signaling promotes male germ cell fate and suppresses female programming in somatic cells. Development.

[63] Souquet B, Tourpin S, Messiaen S, Moison D, Habert R, Livera G (2012). Nodal signaling regulates the entry into meiosis in fetal germ cells. Endocrinology.

[64] Mayère C, Neirijnck Y, Sararols P, Rands CM, Stévant I, Kühne F, Chassot AA, Chaboissier MC, Dermitzakis ET, Nef S (2021). Single-cell transcriptomics reveal temporal dynamics of critical regulators of germ cell fate during mouse sex determination. FASEB J..

[65] Cahoon CK, Hawley RS (2016). Regulating the construction and demolition of the synaptonemal complex. Nat. Struct. Mol. Biol..

[66] Karasu ME, Bouftas N, Keeney S, Wassmann K (2019). Cyclin B3 promotes anaphase I onset in oocyte meiosis. J. Cell Biol..

[67] Souquet B, Abby E, Hervé R, Finsterbusch F, Tourpin S, Le Bouffant R, Duquenne C, Messiaen S, Martini E, Bernardino-Sgherri J, Toth A, Habert R, Livera G (2013). MEIOB targets single-strand DNA and is necessary for meiotic recombination. PLoS Gen..

[68] Dommel S, Blüher M (2021). Does C-C motif chemokine ligand 2 (CCL2) link obesity to a pro-inflammatory state?. Int. J. Mol. Sci..

[69] Schutyser E, Struyf S, Proost P, Opdenakker G, Laureys G, Verhasselt B, Peperstraete L, Van de Putte I, Saccani A, Allavena P, Mantovani A, Van Damme J (2002). Identification of biologically active chemokine isoforms from ascitic fluid and elevated levels of CCL18/pulmonary and activation-regulated chemokine in ovarian carcinoma. J. Biol. Chem..

[70] Furukawa S, Soeda S, Kiko Y, Suzuki O, Hashimoto Y, Watanabe T, Nishiyama H, Tasaki K, Hojo H, Abe M, Fujimori K (2013). MCP-1 promotes invasion and adhesion of human ovarian cancer cells. Anticancer Res..

[71] Iwamoto H, Izumi K, Mizokami A (2020). Is the C-C motif ligand 2-C-C chemokine receptor 2 axis a promising target for cancer therapy and diagnosis?. Int. J. Mol. Sci..

[72] Osgood RS, Upham BL, Hill T, Helms KL, Velmurugan K, Babica P, Bauer AK (2014). Polycyclic aromatic hydrocarbon-induced signaling events relevant to inflammation and tumorigenesis in lung cells are dependent on molecular structure. PLoS ONE.

[73] Fuller EA, Sominsky L, Sutherland JM, Redgrove KA, Harms L, McLaughlin EA, Hodgson DM (2017). Neonatal immune activation depletes the ovarian follicle reserve and alters ovarian acute inflammatory mediators in neonatal rats. Biol. Reprod..

[74] McConnachie LA, Mohar I, Hudson FN, Ware CB, Ladiges WC, Fernandez C, Chatterton-Kirchmeier S, White CC, Pierce RH, Kavanagh TJ (2007). Glutamate cysteine ligase modifier subunit deficiency and gender as determinants of acetaminophen-induced hepatotoxicity in mice. Toxicol. Sci..

[75] NRC (2011). Guide for the Care and Use of Laboratory Animals.

[76] du Sert NP (2020). Reporting animal research: Explanation and elaboration for the ARRIVE guidelines 2.0. PLoS Biol..

[77] Luderer U, Meier MJ, Lawson GW, Beal MA, Yauk CL, Marchetti F (2019). In utero exposure to benzo[a]pyrene induces ovarian mutations at doses that deplete ovarian follicles in mice. Environ. Mol. Mutagen..

[78] Safranski TJ, Lamberson WR, Keisler DH (1993). Correlations among three measures of puberty in mice and relationships with estradiol concentration and ovulation. Biol. Reprod..

[79] Myers M, Britt KL, Wreford NGM, Ebling FJP, Kerr JB (2004). Methods for quantifying follicular numbers within the mouse ovary. Reproduction.

[80] Desmeules P, Devine PJ (2006). Characterizing the ovotoxicity of cyclophosphamide metabolites on cultured mouse ovaries. Toxicol. Sci..

[81] Hirshfield AN (1988). Size-frequency analysis of atresia in cycling rats. Biol. Reprod..

[82] Dobin A, Davis CA, Schlesinger F, Drenkow J, Zaleski C, Jha S, Batut P, Chaisson M, Gingeras TRSTAR (2013). Ultrafast universal RNA-seq aligner. Bioinformatics.

[83] Liao Y, Smyth GK, Shi W (2019). The R package Rsubread is easier, faster, cheaper and better for alignment and quantification of RNA sequencing reads. Nucleic Acids Res..

[84] Nikolayeva O, Robinson MD (2014). edgeR for differential RNA-seq and ChIP-seq analysis: An application to stem cell biology. Methods Mol. Biol..

[85] Eisen MB, Spellman PT, Brown PO, Botstein D (1998). Cluster analysis and display of genome-wide expression patterns. Proc. Natl. Acad. Sci. U. S. A..

[86] Saldanha AJ (2004). Java Treeview–extensible visualization of microarray data. Bioinformatics.

[87] Owa C, Poulin M, Yan L, Shioda T (2018). Technical adequacy of bisulfite sequencing and pyrosequencing for detection of mitochondrial DNA methylation: Sources and avoidance of false-positive detection. PLoS ONE.

[88] Sakurai K, Shioda K, Eguchi A, Watanabe M, Miyaso H, Mori C, Shioda T (2019). DNA methylome of human neonatal umbilical cord: Enrichment of differentially methylated regions compared to umbilical cord blood DNA at transcription factor genes involved in body patterning and effects of maternal folate deficiency or children's sex. PLoS ONE.

