# Supplementary material for: Prenatal exposure to benzo[a]pyrene depletes ovarian reserve and masculinizes embryonic ovarian germ cell transcriptome transgenerationally
Source: Sci Rep. 2023 May 29;13:8671. doi: 10.1038/s41598-023-35494-w (PMC10227008; doi:10.1038/s41598-023-35494-w)

Fig. S1: Primordial, primary and secondary follicle numbers per ovary were significantly decreased in 4-5 month old F2 *Gclm+/-* females descended from pregnant *Gclm+/-* F0 dams dosed daily from E6.5 to E15.5 with 2 mg/kg-day BaP compared to vehicle 0 mg/kg-day controls. *P<0.05, t-test

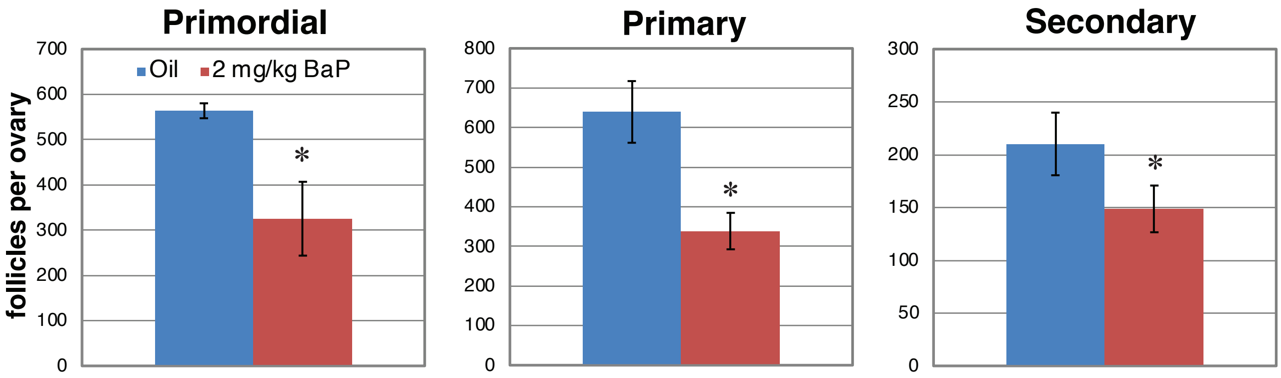

Fig S2: Representative FACS data. P1 and P2 show exclusion of cell debris and clumps. P3 shows gating out of dead cells using propidium iodid (PI) positivity. P4 shows isolation of eGFP-positive germ cells from eGFP-negative somatic cells.

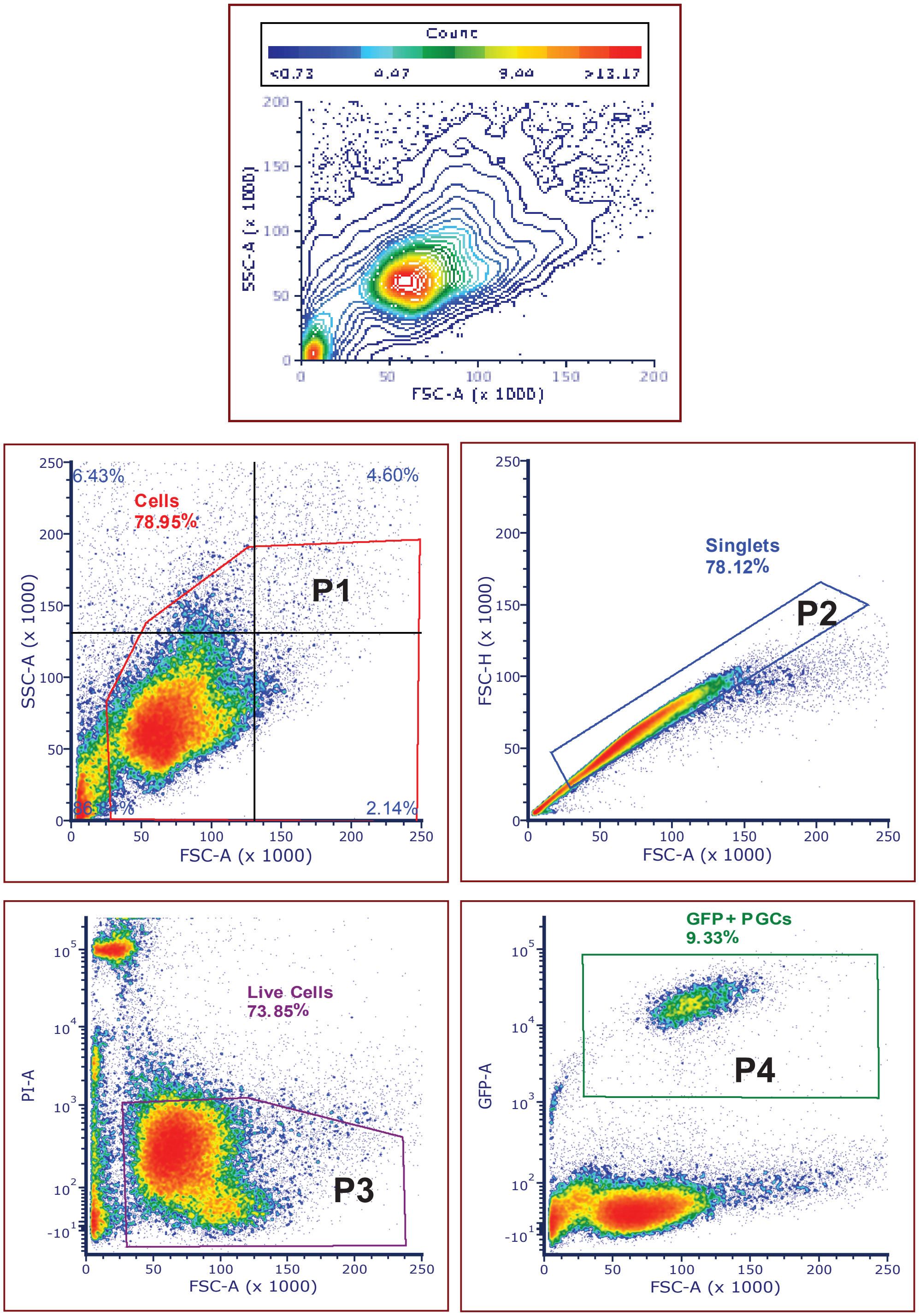

Fig. S3. Primordial Germ Cell RNA-seq: Normalized counts per gene.

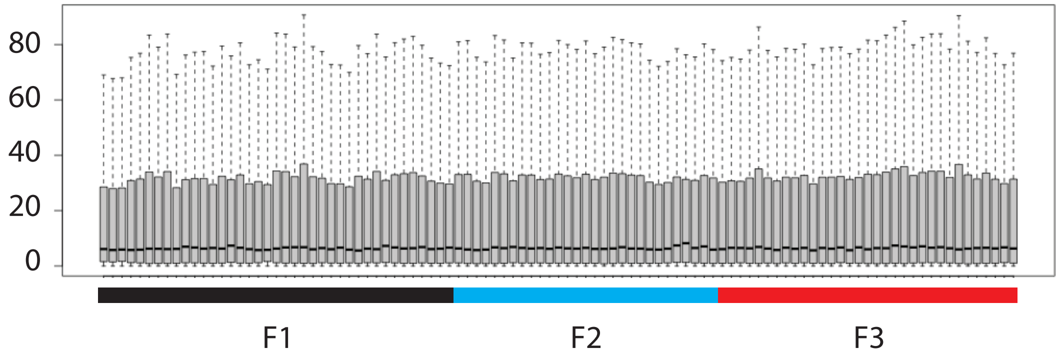

Fig. S4. Primordial Germ Cell RNA-seq: Normalized BigWig tracks of marker genes.

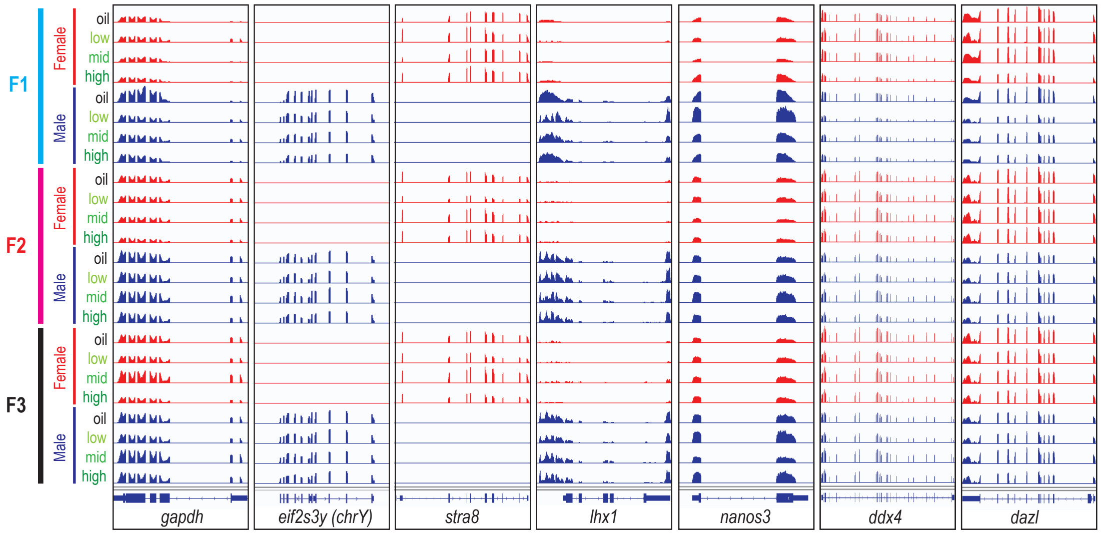

Fig. S5. Permutation Test: Probability of selecting sex-specific DEGs from all expressed genes. Iteration = 5,000. Model fitting = gamma distribution, moment matching estimation

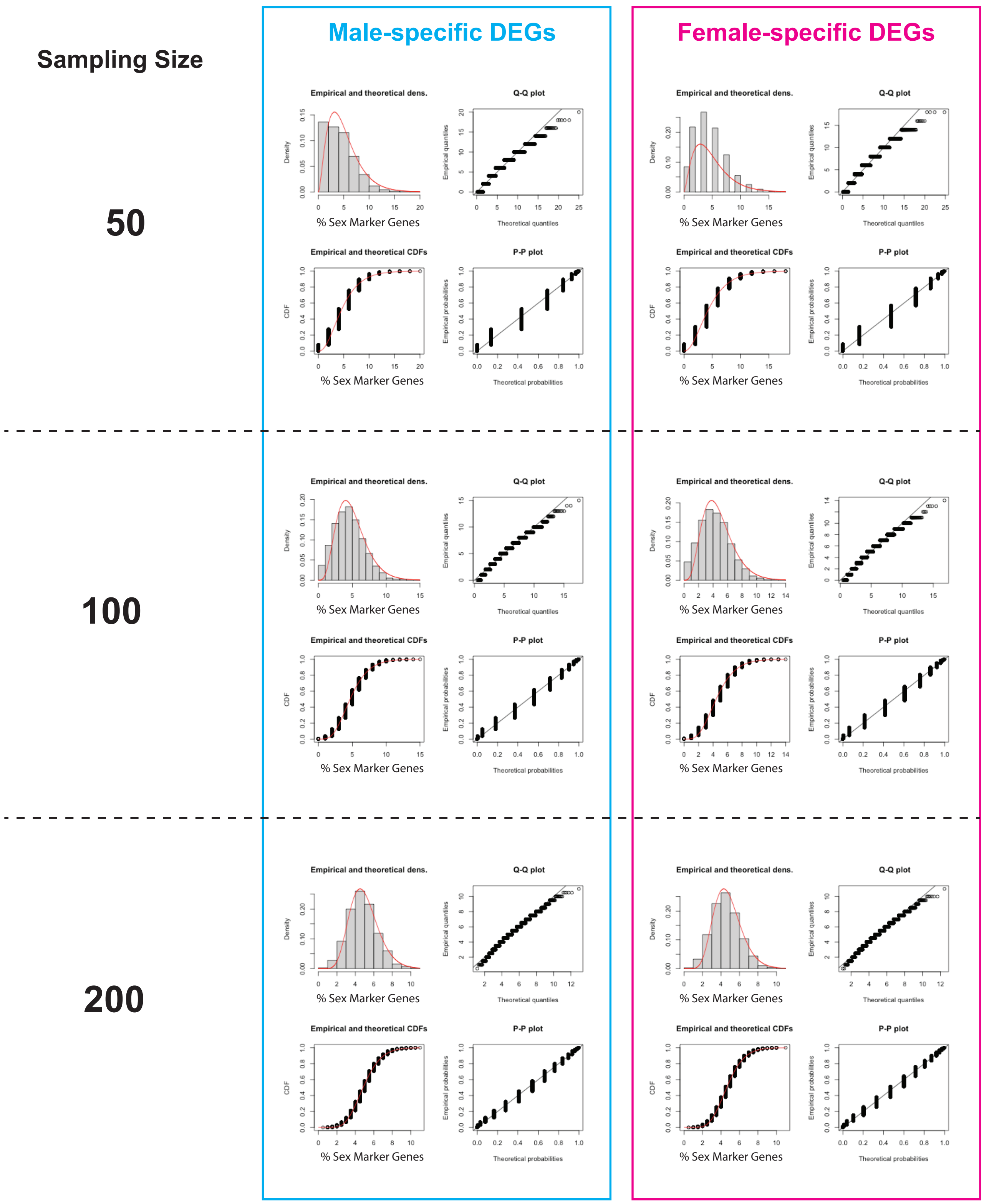

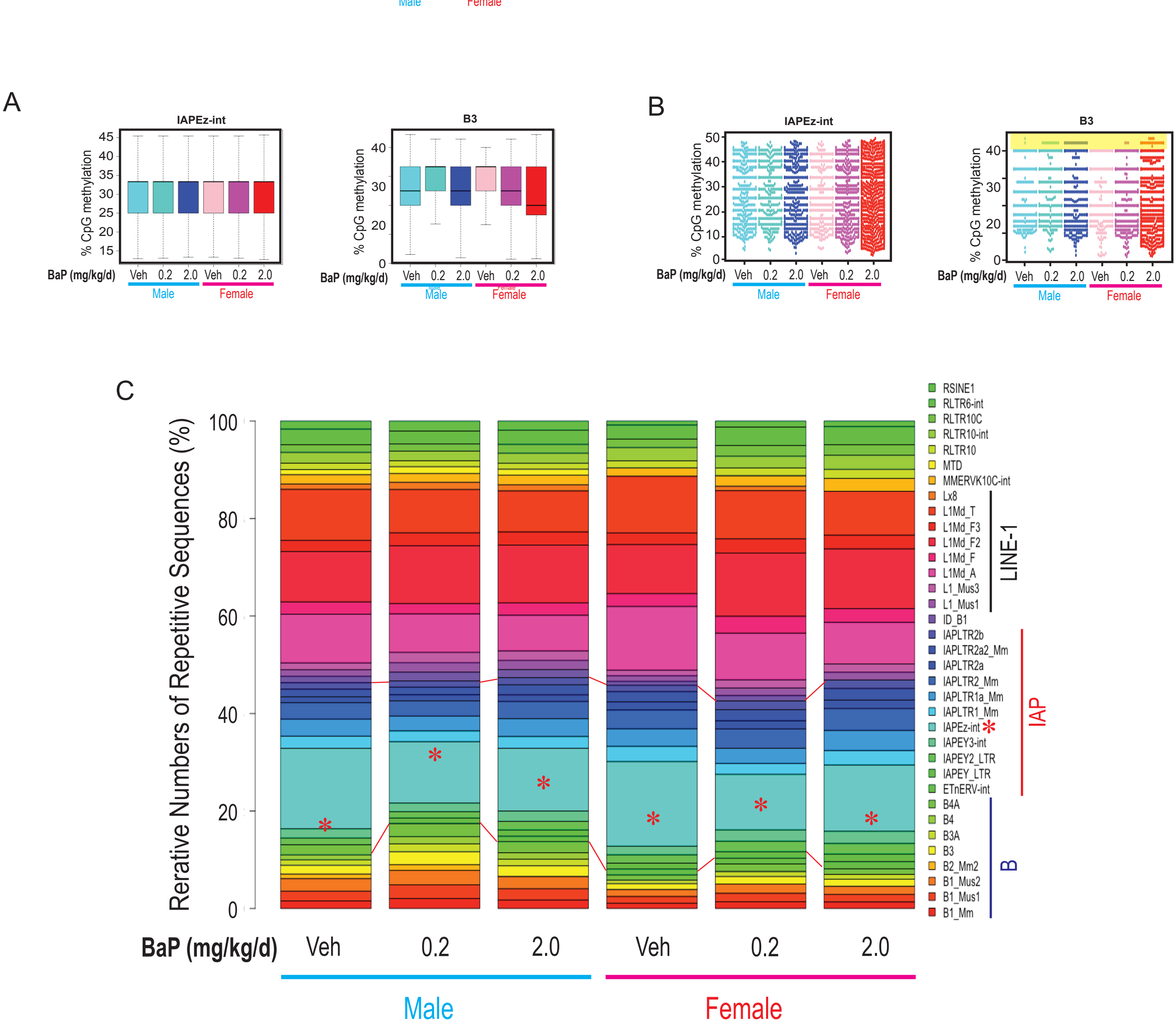

Fig. S6. CpG methylation of the IAPEz-int and B3 repetitive sequences in PGCs isolated from F1 embryos. (A) Boxplots. (B) Beeswarm plots. Highly methylated copies of B3 repeats are shown by yellow shade. (C) Relative numbers of top 35 most strongly methylated repetitive sequences. Asterisk indicates IAPEz-int sequence. Bars indicate LINE-1, IAP, and B1-4 species of repetitive sequences. Experiment shown in Fig. 7D was repeated with an independent set of WGBS data.

Fig. S7. Photomicrographs of hematoxylin and eosin–stained gonad sections from E13.5 embryos. (A) Ovarian germ cells (large and spherical, arrowheads) at E13.5, surrounded by pre-granulosa cells (small and flattened, arrows), show a distinctive pre-meiotic morphology with patches of condensed chromatin at the periphery of the nucleus. (B) Clusters of testicular germ cells (large and spherical, arrowheads) at E13.5 are enclosed by Sertoli cells (irregular in shape, arrows) and a layer of peritubular myoid cells (flattened). Magnification: 400x.

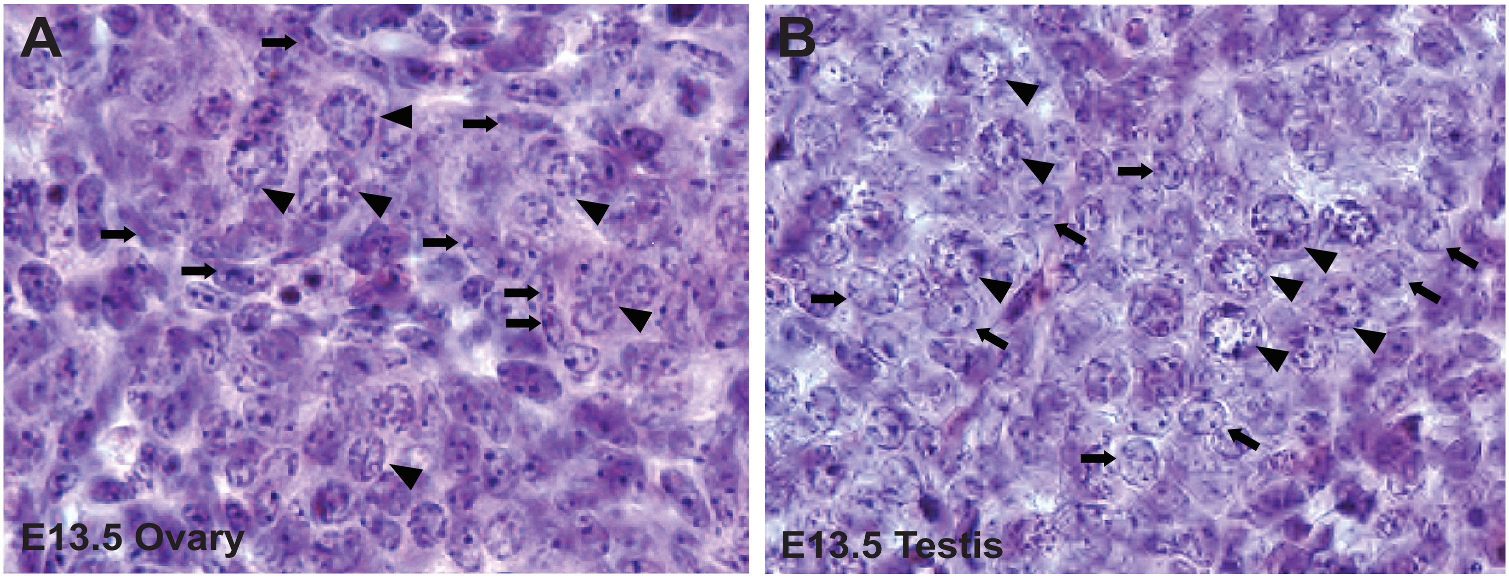

Supplement: Supplementary file 1 — Supplementary Figures. [file 41598_2023_35494_MOESM1_ESM.pdf]
